# Flow-Based Network Analysis of the *Caenorhabditis elegans* Connectome

**DOI:** 10.1371/journal.pcbi.1005055

**Published:** 2016-08-05

**Authors:** Karol A. Bacik, Michael T. Schaub, Mariano Beguerisse-Díaz, Yazan N. Billeh, Mauricio Barahona

**Affiliations:** 1 Department of Mathematics, Imperial College London, London, United Kingdom; 2 naXys & Department of Mathematics, University of Namur, Namur, Belgium; 3 ICTEAM, Université catholique de Louvain, Louvain-la-Neuve, Belgium; 4 Computation and Neural Systems Program, California Institute of Technology, Pasadena, California, United States of America; Hamburg University, GERMANY

## Abstract

We exploit flow propagation on the directed neuronal network of the nematode *C. elegans* to reveal dynamically relevant features of its connectome. We find flow-based groupings of neurons at different levels of granularity, which we relate to functional and anatomical constituents of its nervous system. A systematic *in silico* evaluation of the full set of single and double neuron ablations is used to identify deletions that induce the most severe disruptions of the multi-resolution flow structure. Such ablations are linked to functionally relevant neurons, and suggest potential candidates for further *in vivo* investigation. In addition, we use the directional patterns of incoming and outgoing network flows at all scales to identify flow profiles for the neurons in the connectome, without pre-imposing *a priori* categories. The four flow roles identified are linked to signal propagation motivated by biological input-response scenarios.

## Author Summary

One of the goals of systems neuroscience is to elucidate the relationship between the structure of neuronal networks and the functional dynamics that they implement. An ideal model organism to study such interactions is the roundworm *C. elegans*, which not only has a fully mapped connectome, but has also been the object of extensive behavioural, genetic and neurophysiological experiments. Here we present an analysis of the neuronal network of *C. elegans* from a dynamical flow perspective. Our analysis reveals a multi-scale organisation of the signal flow in the network linked to anatomical and functional features of neurons, as well as identifying different neuronal roles in relation to signal propagation. We use our computational framework to explore biological input-response scenarios as well as exhaustive *in silico* ablations, which we relate to experimental findings reported in the literature.

## Introduction

The nematode *Caenorhabditis elegans* has been used as a model organism in the life sciences for half a century [1], and considerable effort has been devoted to elucidate the properties of its nervous system in relation to functional behaviour. The *C. elegans* connectome was originally charted in 1986 by White *et al* [2] and has been further refined by analysis and experiments [3], most recently in the work of Varshney *et al* [4]. Using experimental techniques such as laser ablations, calcium imaging, optogenetics and sonogenetics, researchers have examined functional properties of individual neurons in connection with motion, learning, or information processing and integration [5–9]. Other studies have quantified the characteristics of the motion of *C. elegans*, and how these change upon genetic mutations [10–12].

With the increased availability of data from such experiments, there is a need to integrate current knowledge about individual neurons into a comprehensive picture of how the network of neurons operates [2, 4, 13]. A number of studies have reported network characteristics of the *C. elegans* connectome: it is a small-world network [14] satisfying mathematical criteria of efficiency [15], with a heavy-tailed degree distribution [16] and a core-set of highly-connected, ‘rich club’ neurons [17]. Furthermore, the analysis of modules in the network has shown that certain strongly coupled clusters of neurons can be linked to biological functions [18–22]. Such observations suggest that a system-wide analysis of the connectome can provide valuable functional information. However, finding simplified mesoscale descriptions that coherently aggregate how information propagates in the directed connectome across multiple scales remains a challenge [23].

In this work, rather than focusing on structural features of the network, we analyse the directed and weighted *C. elegans* connectome from a dynamics-based (more specifically, flow-based) perspective. Using dynamics to probe the relationship between the structure and function of a system has become a valuable tool in many settings [23–25]. In particular, dynamics-based approaches have been successfully used to study brain networks (e.g., fMRI and DSI data [26–28]). For an in depth discussion of network-theoretic methods in neuroscience see the extensive reviews [23, 29, 30]. For an overview on dynamical methods for network analysis we refer the reader to Refs. [24, 25, 31] and the literature cited therein.

Our methods use diffusive processes on graphs as a simple means to link features of the directed network and propagation dynamics. While diffusive flow is a simplification of the actual propagation in the nervous system of *C. elegans*, we can still gain insight into network properties of dynamical interest [4]. We exploit these ideas in two ways. Firstly, we investigate flow-based partitions of the connectome across multiple scales using the Markov Stability (MS) framework for community detection [24, 31, 32]. Our analysis detects subgroups of neurons that retain diffusive flows over particular time scales [33] taking into account edge directionality [24, 34]. We then mimic neuronal ablations computationally, and check *all* possible single and double ablations in the connectome to detect those that are most disruptive of the flow organisation. Secondly, we extract alternative information of the directed network flows through the Role Based Similarity (RBS) framework [35–37]. Without pre-imposing categories *a priori*, RBS classifies neurons into flow roles, i.e., classes of neurons with similar asymmetric patterns of incoming and outgoing network flows at all scales, which are directly extracted from the network. Finally, we mimic ‘stimulus-response’ experiments [5, 7, 38], in which signals propagate through the network starting from well-defined sets of input neurons linked to particular biological stimuli. The ensuing time courses of neuronal flows reveal features of information processing in *C. elegans*, in relation to the obtained flow roles. Our computational analyses are consistent with experimental findings, suggesting that our framework can provide guidance towards the identification of potential neuronal targets for further *in vivo* experiments.

## Results

**Fig 1:**
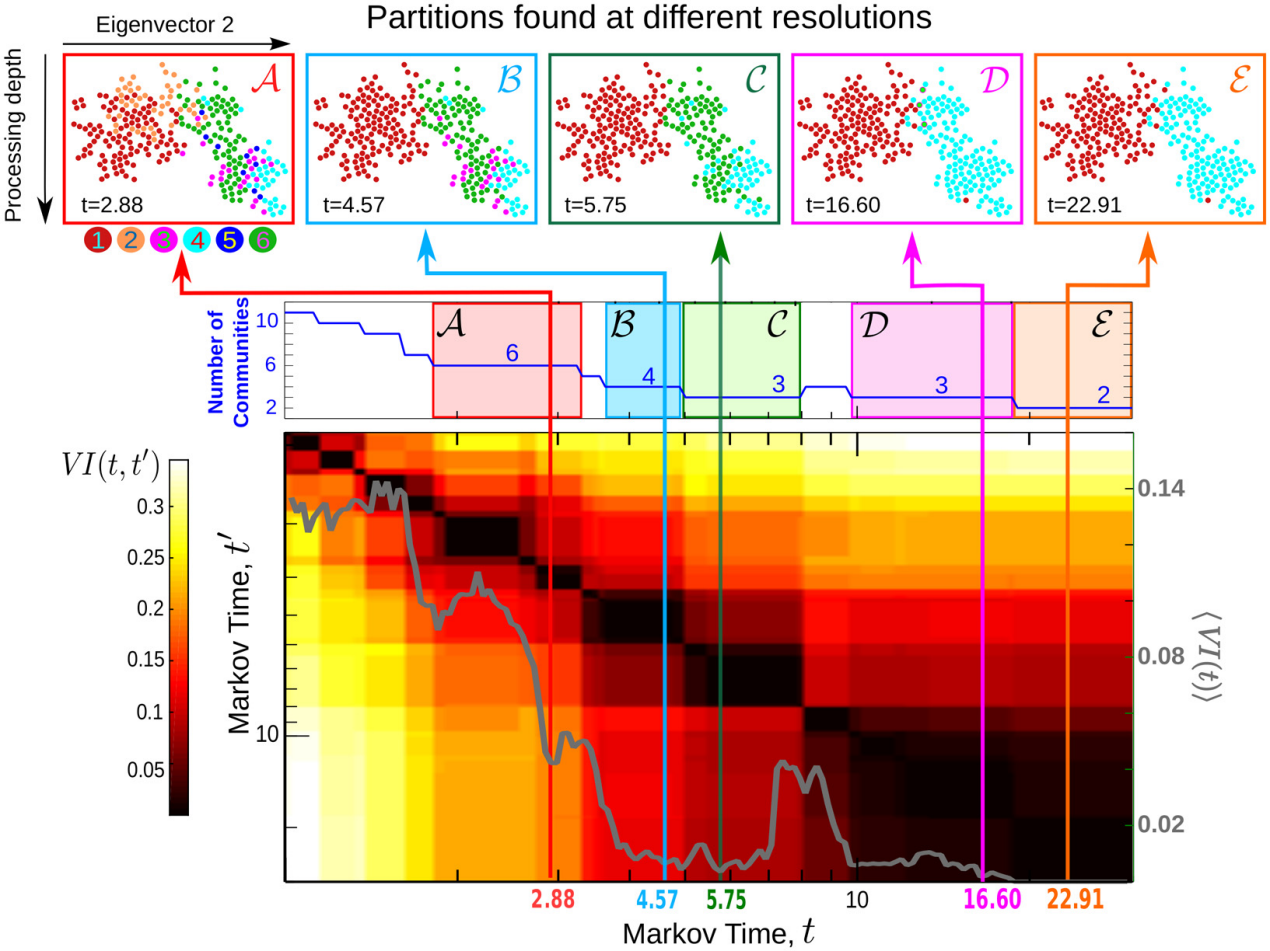
Flow-based multiscale partitioning of the connectome of *C. elegans*. Using Markov Stability, we detect flow-based partitions in this directed network at all scales. Here we show the medium to coarse Partitions A to E (top panel), found as optimal at the indicated Markov time intervals (see S1 Fig for the full sweep of Markov times). The Markov Time intervals corresponding to different robust partitions are indicated by different colour boxes. Partitions A–E are persistent, as signalled by their robustness over extended time plateaux in *VI* (*t*, *t*′)(heatmap in bottom panel), and robust with respect to the optimisation, as signalled by dips in the variation of information 〈*VI*(*t*)〉(grey line in bottom panel).

Our analysis uses the *C. elegans* data published in Ref. [4] (see www.wormatlas.org/neuronalwiring.html). To represent the *C. elegans* connectome, we use the two-dimensional network layout given by [4], i.e., neurons are placed on the plane according to their normalised Laplacian eigenvector (*x*-axis) and processing depth (*y*-axis), as seen in Fig 1 (top panel). We study the largest weakly-connected component of this network, which contains 279 neurons with 6394 chemical synapses (directed) and 887 gap junctions (bidirectional). Reference [4] also provides the position of the soma of each neuron along the body of the worm, and classifies each neuron as either sensory (S), interneuron (I) or motor (M).

### Flow-based partitioning reveals multi-scale organisation of the connectome

To reveal the multi-scale flow organisation of the *C. elegans* connectome, we use the Markov Stability (MS) framework described in Sec. ‘A dynamical perspective for community detection in graphs: Markov Stability’. Conceptually, MS can be understood as follows. Imagine that a drop of ink (signal) is placed on a node and begins to diffuse along the edges of the graph. If the graph lacks structural organisation (e.g., random), the ink diffuses isotropically and rapidly reaches its stationary distribution. However, the graph might contain subgraphs in which the flow is trapped for longer than expected, before diffusing out towards stationarity. These groups of nodes constitute dynamical, flow-retaining communities in the graph, usually signifying a strong dynamic coherence within the group and a weaker coherence with the rest of the network. If we allow the ink to diffuse just for a short time, then only small communities are detected, for the diffusion cannot explore the whole extent of the network. If we observe the process for a longer time, the ink reaches larger parts of the network, and the flow communities thus get larger. By employing dynamics, and in particular by scanning across time, MS can thus detect cohesive node groupings at different levels of granularity [24, 33, 39]. In this sense, the time of the diffusion process, denoted *Markov time* in the following, acts as a resolution parameter.

**Fig 2:**
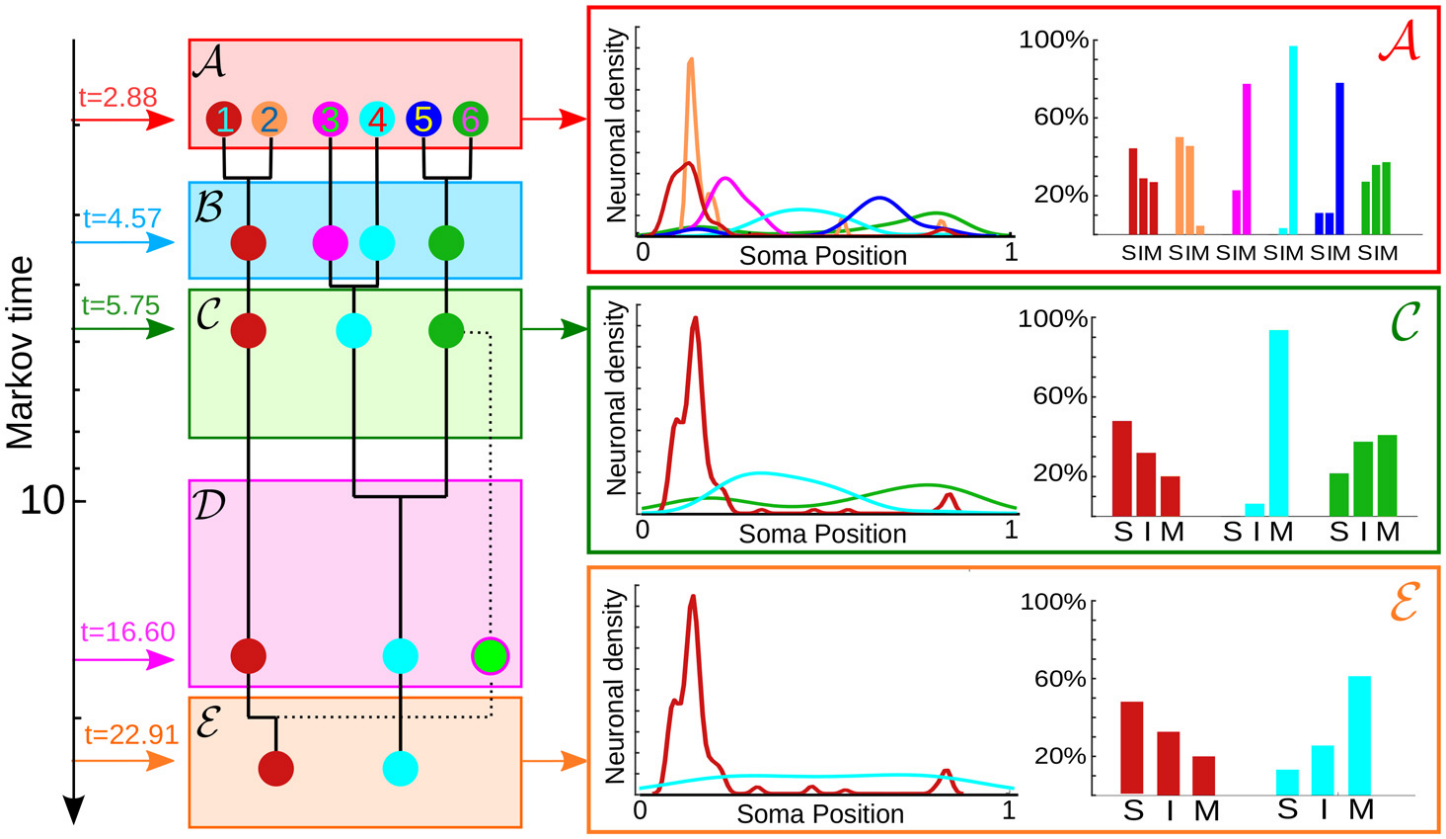
Community structure and biological features. Left: As indicated by the dendrogram, the partitions obtained have a quasi-hierarchical organisation. The dotted line indicates that the light green community at *t* = 16.60 does not result from a hierarchical merging. Middle: The smoothed spatial densities of neurons in each community for the different partitions show how the communities are spatially grouped according to soma positions along a longitudinal axis normalised between 0 and 1. The merging of groups over Markov time largely retains this spatial structure. Right: The percentages of sensory (S), inter- (I) and motor (M) neurons in each community show functional segregation in the groupings.

The flow-based community structure of the *C. elegans* connectome at medium to coarse levels of resolution is shown in Fig 1. The full scan across all Markov times is shown in S1 Fig and the S1 Dataset. As described above, the partitions become coarser as the Markov time *t* increases, from the finest possible partition, in which each node forms its own community, to the dominant bi-partition at long Markov times. The sequence of partitions exhibits an *almost hierarchical* structure, with a strong spatial localisation linked to functional and organisational circuits (see Fig 2 and S2 Fig). These findings are in agreement with the spatial localisation of functional communities often found in brain networks [23], as well as the hierarchical modularity exhibited by the *C. elegans* connectome as reported in Ref. [40]. We remark that our community detection method does not enforce a hierarchical agglomeration of communities: the observed quasi-hierarchy and spatial localisation is an intrinsic feature of the *C. elegans* connectome. In S2 Fig we quantify the deviation of the community structure from a strict hierarchy.

At long Markov times, we find robust partitions containing 6 to 2 communities, denoted A to E in Fig 1. Partition A comprises six communities of varying sizes (from 9 to 104 neurons), well localised along the body of the worm, as seen in Fig 2 (c.f. Section 2.2 in www.wormatlas.org/neuronalwiring.html). The two large communities (A1 and A2) have head ganglia neurons of all three functional types (S, I, M). In particular, A1 contains ring motor neurons and interneurons as well as the posterior neurons ALN and PLN, whereas A2 specifically gathers amphid neurons (e.g., AWAL/R, ASKL/R, ASIL/R, AIYL/R) which feature prominently in the navigation circuit responsible for exploratory behaviour [41]. Communities A3, A4 and A5 in Partition A consist predominantly of ventral cord motor neurons, differentiated by their soma position along the body (Fig 2): A3 contains frontal motor neurons (e.g. VD1 to VD3); A4 consists of mid-body motor neurons (e.g. VD4 to VD8); A5 comprises posterior motor neurons (e.g. VD9 and VD10). Such partitioning is consistent with the motor neuron segmentation model proposed for *C. elegans* in Ref. [42]. Finally, A6 contains highly central neurons such as AVAL/R or PVCL/R, which have been found to belong to a *rich-club* [17], as well as interneurons linked to mechanosensation and tap withdrawal functional circuits [20].

The coarser Partitions B and C are quasi-hierarchical merges of A (Figs 1 and 2). For instance, Partition C has three groupings: head ganglia (merged A1 and A2), frontal motor neurons (merged A3 and A4), and a tail subgroup (merged A5 and A6). Interestingly, at later Markov times, we obtain the distinct, coarser 3-community Partition D, which exemplifies how our method does not enforce a strict hierarchy in the multiscale structure. The three groups in Partition D include a notable community of only three nodes (interneurons AVFL/R and AVHR), which appear as a cohesive group only at this particular timescale. Prominent functional roles of AVF and AVH neurons have been noted previously [4, 43]: both AVF neurons are responsible for coordination of egg-laying and locomotion [44]. In addition, spectral analyses of the gap-junction Laplacian have shown that AVF, AVH, PHB and C-type motor neurons are strongly coupled [4]. Finally, the two communities in the coarsest Partition E split the connectome anatomically into a group with head and tail ganglia (red), and another group predominantly with motor neurons (cyan).

### The effect of single and double neuron ablations on flow-based communities

Laser ablation experiments are invaluable to probe the functional role of neurons [5–7], but are time consuming and technically challenging. We have used our computational framework to assess the effect that an ablation of a single neuron, or of a pair of neurons, has on the signal flow in the connectome. To this end, we compare the flow-based partitions obtained for the ablated connectome against the original network. If an ablation creates large distortions in the flow structure, the partitions of the ablated network will change drastically or become less robust compared to those found in the unablated network. We have carried out a systematic computational analysis of *all* single and double neuron ablations in the connectome.

#### Single ablations: Disrupting the robustness and make-up of partitions

**Fig 3:**
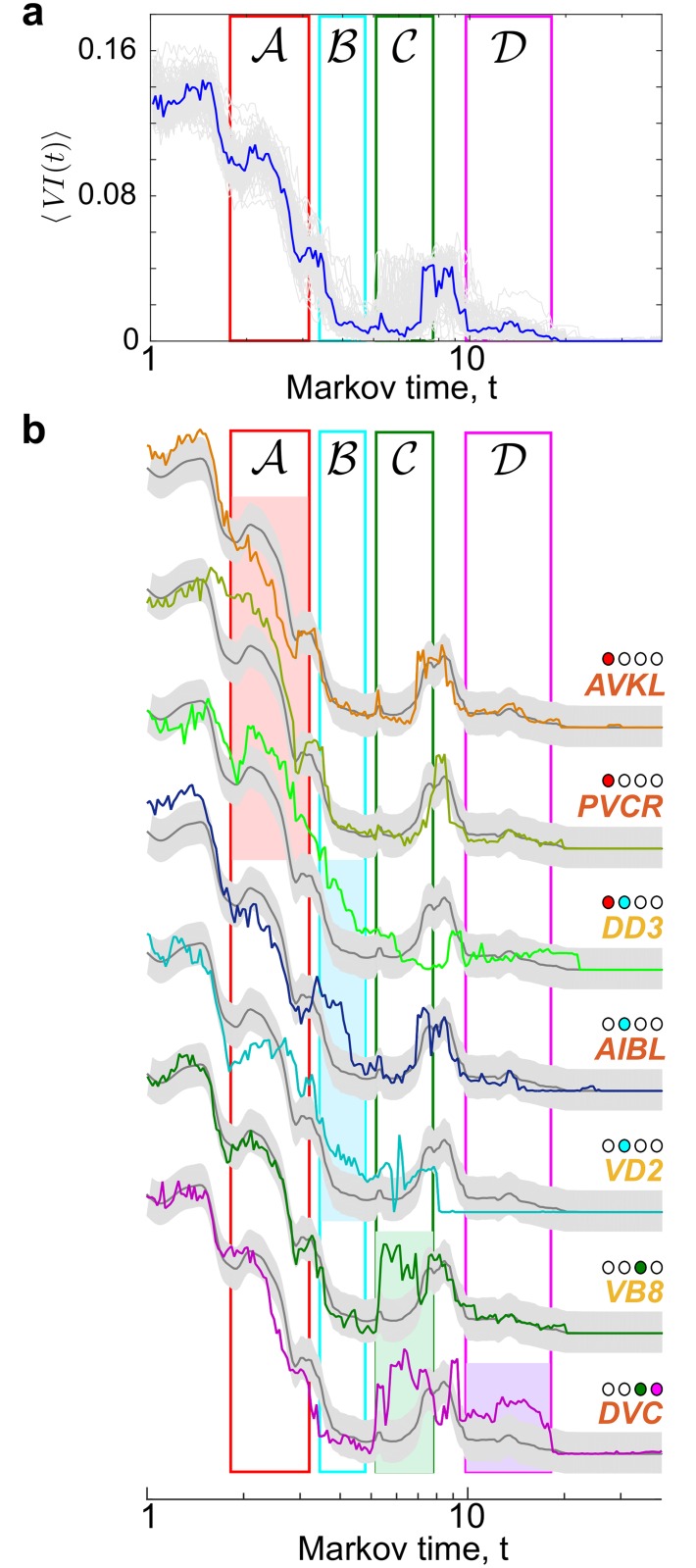
Single ablations that alter the robustness of partitions. **(a)** Ensemble of 〈*VI*(*t*)〉 profiles of *all* single node ablations (light gray lines) and the unablated connectome (blue). A Gaussian process (GP) is fitted to the ensemble of single ablations. **(b)** The GP is described by the mean *μ*(*t*) (dark grey line) and standard deviation (grey bands). Sustained outliers from the GP are identified using a statistical criterion to find seven ablations that affect the different partitions, as indicated by the coloured dots.

*Ablations that alter the robustness of partitions.* To find ablations that have a strong effect on the robustness of Partitions A–E, we detect node deletions that induce sustained changes in the robustness 〈*VI*(*t*)〉, i.e., they appear as outliers with respect to a Gaussian Process fitted to the 〈*VI*(*t*)〉of the ensemble of *all* single node ablations (Fig 3). For details, see Section ‘Quantifying the disruption of community structure under node deletion’.

Only seven single ablations satisfy our criterion for a major disruption of any of the Partitions A–E (Fig 3b). The ablations of interneuron PVCR or of the motor neuron DD3 both decrease the robustness of Partition A. Interestingly, PVCR (A6) and DD3 (A4) receive many incoming connections from their own community. Furthermore, both of these neurons are critical for motor action: PVCR drives motion whereas DD3 coordinates it. Another important ablation is that of interneuron AVKL, which links community A1 (head) with community A3 (ventral cord) and community A6 (rich club). The increased robustness of the community structure upon ablation of AVKL would indicate a decreased communication between these groups. The function of AVKL is uncharted at present [45], suggesting further *in vivo* experimental investigations to explore any behavioural changes as a result of its ablation.

There are three important ablations in Partition B: DD3 (again), VD2 (another D-type motor neuron yet on the ventral side), and AIBL, an amphid interneuron. AIBL acts as a bridge between communities A1 and A2, which merge in Partition B (Fig 2). The prominent role of other amphid interneurons will become apparent in the double ablations studied in the next section.

Partition C is rendered non robust by the ablations of VB8 (a motor neuron responsible for forward locomotion) or of interneuron DVC, with are both in community A5. DVC has links with communities A3, A4, A5 and A6; hence its ablation affects the subsequent merging of these groups. Note that the ablation of DVC reduces the robustness of both 3-way Partitions C and D, thus blurring the spatial organisation of motor neurons. This indicates that DVC might integrate feedback from different parts of the body, in accordance with the fact that it has the highest number of gap junctions in the connectome, as well as substantial chemical synapses [46].

Our study of ablations that affect the robustness of partitions can be linked to the study of ‘community roles’ [47]. Using such categorisation, the neurons mentioned above are classified as either connector or provincial hubs (e.g., DVC is a ‘non-hub’ connector node) [20].

**Fig 4:**
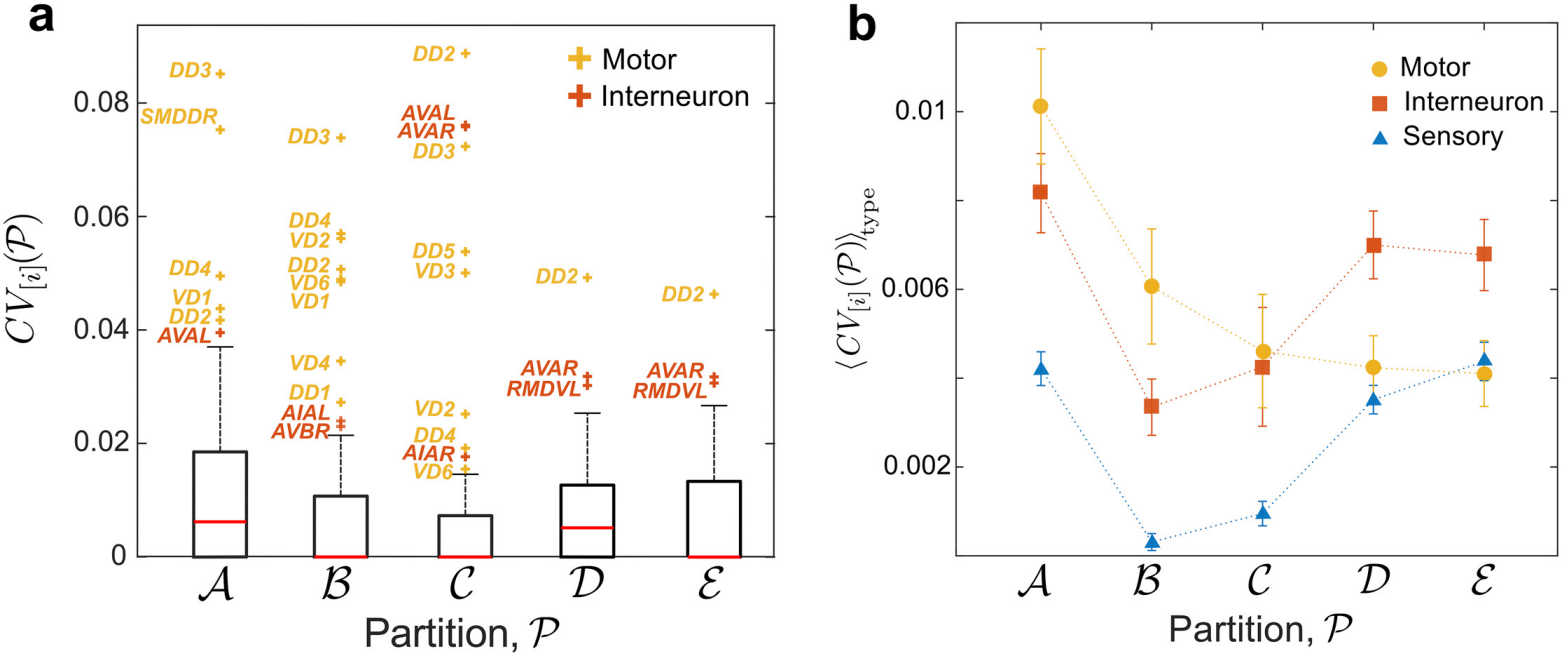
Effect of single ablations on the make-up of different partitions as measured by the community variation. (**a**) The disruption of every single mutation with respect to Partitions A–E is quantified through $CV_{\left[i\right]}\left(P\right)$, as defined in Eq (14). The distribution of *CV*_[*i*]_ is represented by its median (red line) and the inter-percentile range (IPR) between the 10th and 90th percentiles (box). The whiskers correspond to the IPR for each partition, and the single ablations detected as outliers are labelled. (**b**) Effect of the single ablations $CV_{\left[i\right]}\left(P\right)$ for each partition averaged over each type: sensory (blue), inter- (red) and motor neurons (yellow). On average, single ablations of motor neurons induce larger changes on the finer Partitions A and B, whereas ablations of interneurons have a larger effect on the coarser Partitions D and E. The error bars are the standard error of the mean.

*Ablations that alter the make-up of the optimal partitions.* To measure how much the make-up of a partition is affected by an ablation, we use the community variation *CV*, defined in Eq (14). A high value of $CV_{\left[i\right]}\left(P\right)$ indicates a large disruption in partition P under the ablation of neuron *i*. Fig 4 shows the single ablations with high *CV* with respect to Partitions A–E, as detected through a statistical criterion based on interpercentile ranges (see Section ‘Quantifying the disruption of community structure under node deletion’). Interestingly, none is a sensory neuron, indicating that the ablation of sensory neurons is not influential for global flow at medium to coarse scales, although they can have strong local effect on the propagation of a particular stimulus.

Certain ablations are completely destructive of Partitions A and B. In particular, the ablations of DD3 or SMDDR induce severe changes in the network flow, so that no partition similar to A is found at any Markov time. In general, ablations of D-type motor neurons coordinating motion (e.g. DD2, DD3, VD1, VD2) have particularly severe effects for the medium resolution Partitions A and B. Interestingly, D-type motor neurons have significantly higher PageRank (median 0.0092 compared to median of 0.0018 in the network; *p* = 1.7 × 10^−7^; one-sided exact test), and their synapses are critically embedded edges with few alternative routes [43]. Note that, although robustness and make-up of partitions reflect different effects, the ablation of motor neurons DD3 and VD2 substantially alters both (see Figs 3 and 4a). In addition, the ablation of any of the command neurons AVAR/L has important effects on Partition C. AVAR/L are highly central neurons (with the highest in- and out- degree in the connectome) and our method confirms that their ablation introduces heavy distortions in the global flow of the connectome. Finally, we observe that the coarsest partitions D and E are strongly perturbed upon ablation of ring motor neuron RMDVL. Experiments have shown that ablating any of the RMD neurons diminishes the head-withdrawal reflex [1].

Further confirmation of the importance of inter- and motor neurons is given in Fig 4b, where we show the *CV* of single ablations averaged over the three types (S, I, M). On average, motor neurons tend to have a stronger effect on local organisation due to their localised connectivity; this is reflected by the high *CV* in the finer Partitions A and B. On the other hand, interneurons, which are mediators of information flow from sensory to motor neurons, can induce large changes in global flows, as shown by larger *CV* for the coarser Partitions D and E.

#### Double ablations: Beyond additive effects

We have also performed an exhaustive *in silico* exploration of all possible 38781 two-neuron ablations. Specifically, we look for synergistic pairs of neurons, i.e. pairs whose simultaneous ablation induces supra-additive disruption. To this end, we compare the *CV* for each double ablation to the averaged *CV* of the corresponding two single ablations, and use Quantile Regression to identify double ablations with a combined effect significantly beyond the merely additive (see Section ‘Detecting supra-additive double-node deletions’).

**Fig 5:**
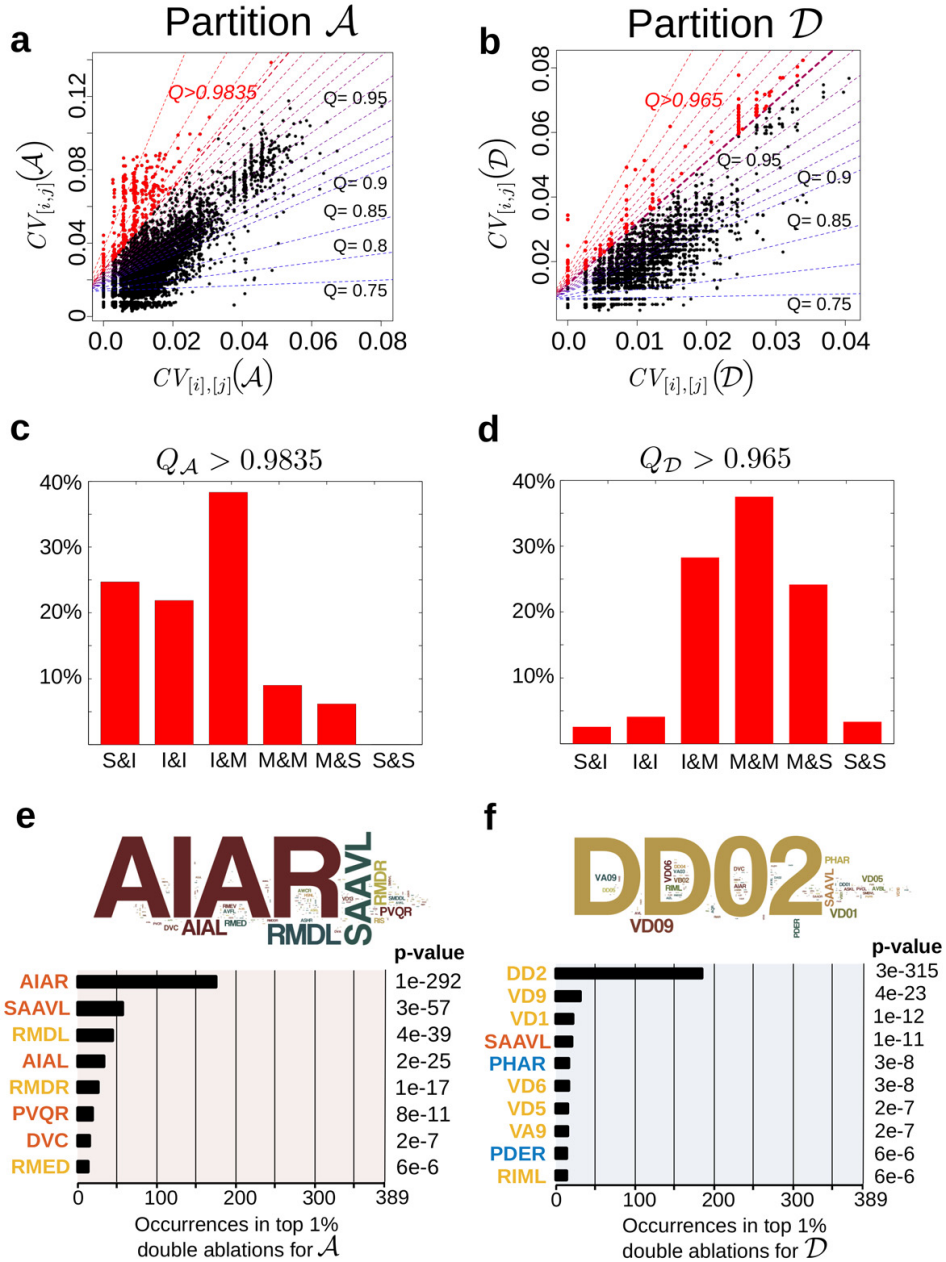
Supra-additive double ablations. The combined effect of each two neuron ablation is compared against the additive effect of the corresponding two single ablations. The results of Quantile Regression of *CV* of the pair against the averaged *CV* of the two single ablations (see Section ‘Detecting supra-additive double-node deletions’) are shown for: (**a**) Partition A and (**b**) Partition D. The top 1% pairs with the largest supra-additive effect are found above the quantile scores $Q_{A}>0.9835$ and $Q_{D}>0.965$, respectively. These top 1% double ablations are dominated by: **(c)** interneurons for A; **(d)** motor neurons for D. Overrepresentation of neurons in the top 1% supra-additive pairs for **(e)** Partition A and **(f)** Partition D was calculated using a one-sided Fisher exact test (unadjusted p-values are reported, and also provided for all neurons in the S1 Dataset). Neurons with *p* < 10^−5^ are listed and the names of neurons are coloured according to their type: S (blue), I (red), M (yellow). The word clouds are a visualisation of these over-representations. Computing a Bayesian quantile of higher prevalence of these neurons among the top 1% pairs also supports these findings [48].

We focus on disruptions to Partitions A and D, as prototypical of the medium and coarse resolutions, respectively (Fig 5). We select the top 1% of ablations for each partition according to their supra-additive effect. Interestingly, 85% of the top supra-additive double ablations for Partition A contain at least one interneuron, whereas 90% of the top supra-additive double ablations for Partition D contain at least one motor neuron (Fig 5c and 5d). This observation complements the results for single ablations in Fig 4. For Partition A, maximal impact of a single ablation is achieved through the deletion of motor neurons, but double ablations containing interneurons are more synergistic. For the coarser Partition D, the most disruptive single ablations are those of interneurons, yet on average the most synergistically disruptive double ablations include motor neurons. Such joint effects underline the structured complexity of the connectome network and reinforce the fundamental importance of I and M neurons in the disruption of flows. In particular, the relative abundances of particular neurons in the top supra-additive pairs (Fig 5e and 5f) show that interneurons AIAR/L, SAAVL and PVQR and motor neurons RMDL/R are overly represented for Partition A. These neurons thus have a magnifying disruptive effect for the medium scales of the connectome. For the coarser Partition D, this magnifying effect on larger scales is induced mostly by motor neurons DD2, VD9, VD1 and interneuron SAAVL.

If we consider the effect on both medium and large scales, only nine double ablations appear in the top 1% for both partition A and D (Table 1). Interestingly, none of these pairs is linked by an edge in the connectome. Note that eight out of these nine pairs contain interneuron AIAR. The amphid interneurons AIA (along with AIB, AIY and AIZ) have a specific position in the connectome: they receive synapses from sensory neurons driving motion. Their prominent role in locomotion integration has been previously discussed and backed by *in vivo* ablation experiments [6]. Our results indicate that the deletion of pairs of neurons involving AIAR would have a particularly magnifying effect on the disruption of the flow organisation at all scales in the connectome. Note that the effect of AIAL in double ablations is much less prominent. The asymmetry observed in how the ablations of AIAR and AIAL affect the flows in the connectome is worth of further experimental investigation. The full set of outcomes of both single and double ablations are presented in S1 Data as a guide for possible experimental investigations.

**Table 1:** Double ablations within the top 1% of supra-additive pairs for both Partition A and D chosen according to their quantile scores ($Q_{A}>0.9835$ and $Q_{D}>0.965$).

Double ablation	Neuron types	$Q_{A}$	$Q_{D}$
AIAR + AQR	I & S	0.9975	0.9785
AIAR + AVEL	I & I	0.9975	0.9650
AIAR + DA2	I & M	0.9875	0.9785
AIAR + VA2	I & M	0.9945	0.9655
AIAR + VB2	I & M	0.9970	0.9785
AIAR + VD5	I & M	0.9950	0.9785
AIAR + VD6	I & M	0.9950	0.9785
AIAR + PVCR	I & I	0.9980	0.9785
SAAVL + AQR	I & S	0.9955	0.9730

### Identifying flow profiles in the directed connectome

A complementary analysis of the directed connectome of *C. elegans* is provided by the Role Based Similarity (RBS) framework [35, 36], which identifies groups of nodes with similar *flow profiles* in the network without imposing *a priori* the type or number of groups. Such groups of neurons display the same character (or *flow role*) in terms of their role in the generation, distribution and consumption of flow in the network. Briefly, RBS obtains a *flow profile* for each node from its incoming and outgoing flows at all scales. We then group the nodes into classes (‘flow roles’) with similar in- and out-flow patterns. Because they include information at all scales, flow roles capture nuanced information about the network, beyond pre-defined categories (e.g., sources, sinks, hubs) or combinatorial notions based on immediate neighbourhoods (e.g., roles from Structural Equivalence [49] and Regular Equivalence [50]). Details of the RBS methodology are given in Refs. [34–37], and summarised in Section ‘Finding flow roles in networks: Role-based similarity’ and in the S4 Fig.

**Fig 6:**
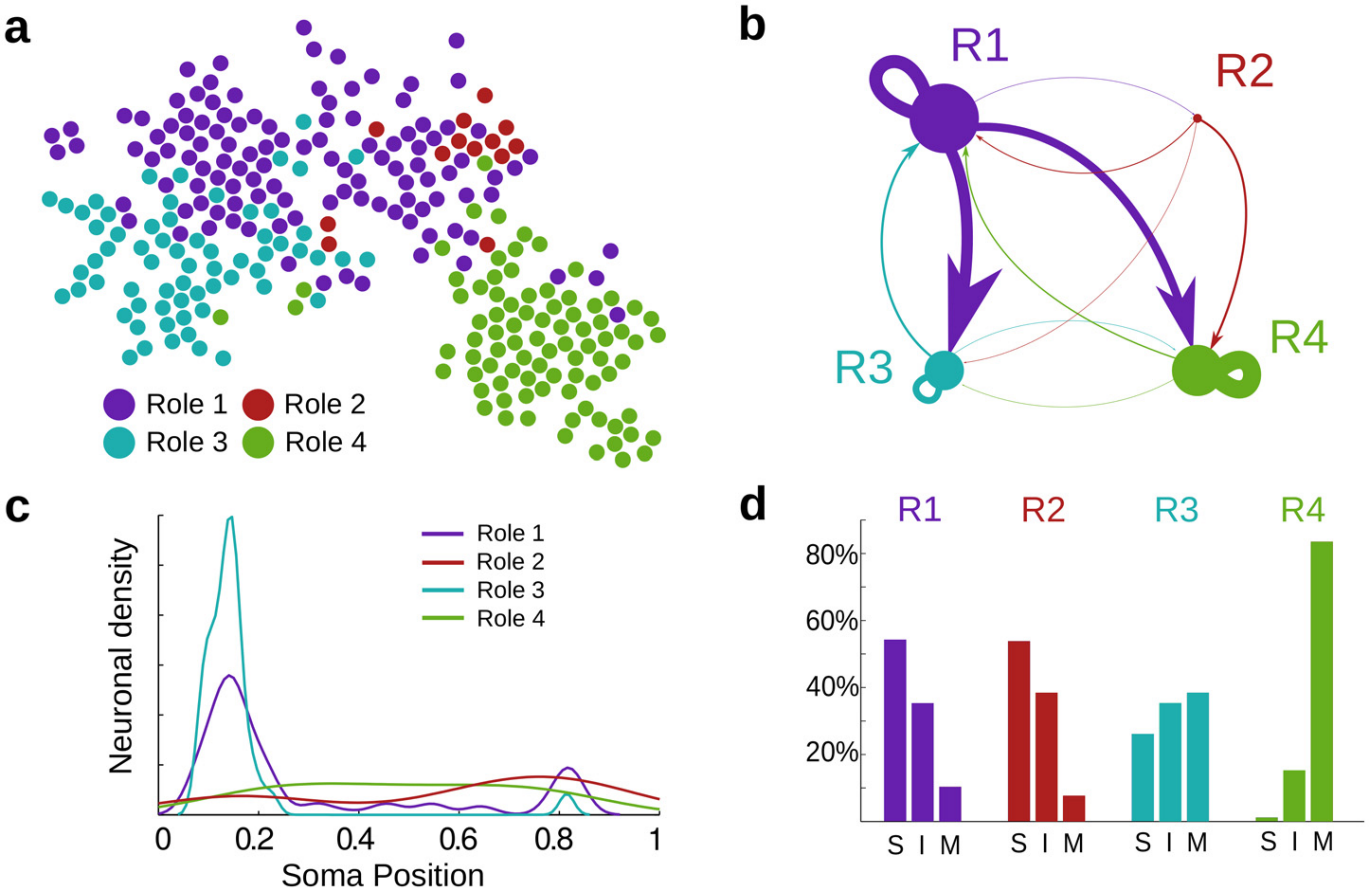
Flow roles for neurons in the *C. elegans* connectome. (**a**) Using RBS, we detect four flow roles in the directed connectome. (**b**) The coarse-grained representation summarises the flow profiles of the roles: two upstream roles (R1, R2), with a dominant source character and high PageRank (S6 Fig), and two downstream roles (R3, R4), with a dominant sink character and lower PageRank. Yet each role has distinctive in- and out-flow patterns in relation to the others. (**c**) Spatial density of neurons for each flow role represented as a function of the normalised soma position: R1 and R3 are localised predominantly in the head; R2 and R4 are spread out along the body. Note how the upstream flow role R2 has noticeable localisation in the tail. (**d**) The percentages of sensory (S), inter- (I) and motor neurons (M) in each role underline their functional differences.

In the *C. elegans* connectome, we identify four distinct classes of neurons according to their flow profiles (Fig 6). These flow roles are distinct from the groupings into communities (see an analysis of communities and their mix of flow roles in the S5 Fig). Two of the roles (R1 and R2) have a dominant ‘source’ character (i.e., higher average in-degree than out-degree) and contain most of the nodes with high PageRank (S6 Fig). The other two roles (R3 and R4) have a dominant ‘sink’ character and nodes with low PageRank. Note, however, that these roles are not just defined by average properties, but by their global flow patterns in the network. As seen in Fig 6b, R1 is upstream from R3 and R4, whereas R2 is mostly upstream from R4. Furthermore, R4 is an almost pure downstream module, whereas R3 has a stronger feedback connection with R1.

The RBS flow roles are linked to physiological properties of the neurons (Fig 6c and 6d). R4 corresponds to a group of motor neurons (mostly ventral chord motor neurons) consistent with its downstream character, whereas R1 is a group of mostly sensory and inter-neurons with heavy localisation in the head. R3 is a group with a balanced representation of all three types of neurons (including some polymodal neurons) localised in the head. Indeed, most ring neurons in R3 are in community A1, indicating a self-contained unit that process head-specific behaviour, such as foraging movements and the head withdrawal reflex [45].

Our RBS analysis also reveals a specific flow profile (R2) containing 13 neurons (mainly sensory and interneurons, mostly upstream from the motor neurons in R4), the majority of which are responsible for escape reflexes triggered in the presence of noxious factors (Table 2). This group can be seen as a group of *escape response neurons* and include: the PVDL/R neurons, which sense cold temperatures and harsh touch along the body; FLPL/R, which perform the equivalent task for the anterior region; PHB neurons responsible for chemorepulsion; PHCR, which detects noxiously high temperatures in the tail; SDQL and PQR, which mediate high oxygen and CO_2_ avoidance, respectively; and PLMR, a touch mechanosensor in the tail [2]. This escape response group is heavily over-connected to command neurons AVAL/R, AVDL/R, DVA, PVCL/R, all of which modulate the locomotion of the worm. (Specifically, there are 48 connections from R2 to these particular command neurons in contrast to the ∼12 connections expected at random.) Note that AVDL/R and DVA are in R1, whereas AVAL/R and PVCL/R are in R4; the R2 group thus links directly to motor locomotion neurons across the worm. We remark that this group of neurons was found exclusively through the analysis of their all-scale in/out flow profiles, without any other extrinsic information.

**Table 2:** Role 2 (R2) neurons: The thirteen neurons identified in R2 constitute a group of *escape response neurons* containing mostly sensory and inter-neurons linked with escape reflex reactions in response to different noxious stimuli.

Neuron(s)	Noxious factor
FLPL/R	Harsh touch, low temperature (head)
PHBL/R	Chemicals
PHCR	High temperature (tail)
PLMR	Gentle touch (tail)
PQR	CO_2_
PVDL/R	Harsh touch, low temperature
SDQL	High O_2_
SAAVL/R	No known factor
VD11	No known factor

### Information propagation in the connectome: Biological input scenarios

Despite its modest size, the nervous system of *C. elegans* can sense and react to a wide range of mechanical, chemical and thermal factors [45]. Standard notions in neuroscience hold that stimuli lead to motor action due to information progressing from sensory through inter- to motor neurons [51]. However, the underlying mechanisms and precise signal flows are still far from understood. In the absence of measurements probing such pathways, and as a first approximation to more realistic nonlinear dynamical models, we use here simplified diffusive dynamics (see Section ‘Propagation dynamics in the network’) to mimic signal propagation in the *C. elegans* directed network. Such an approach, already suggested by Varshney *et al*. [4], is naturally linked to MS multiscale community detection and to the identification of RBS flow roles, since both Markov Stability and Role Based Similarity are intrinsically defined in terms of a diffusive process on the graph.

To mimic the propagation of stimuli associated with particular biological scenarios, a normalised initial flow vector ***ϕ***(0) is localised at specific input neurons and we observe the decay towards stationarity under Eq (5):

θ(t)=ϕ(t)-π. (1)

We also define ***q***(*t*), which will be used to detect overshooting neurons:

$$q_{i}\left(t\right)=\frac{\phi_{i}\left(t\right)}{\pi_{i}}=1+\frac{\theta_{i}\left(t\right)}{\pi_{i}}.$$ (2)

Initially, *θ*_*i*_(0) is positive only for the input neurons where we inject the signal, and negative for all other neurons. Asymptotically, the vector of flows ***ϕ***(*t*) approaches the stationary solution ***π***, and *θ*_*i*_(*t*) → 0, ∀*i*. However the approach to the stationary value can be qualitatively different. In some cases, *θ*_*i*_(*t*) can become positive, if neuron *i* receives an influx of flow that drives it to ‘overshoot’ above its stationary value; in other cases, neurons approach stationarity without overshooting. The different behaviour depends on the particular initial input and the relative location of each neuron in the network.

Motivated by several experimental studies, we have conducted four case studies corresponding to different biological scenarios in which the input is localised on specific neurons:

(i1) Posterior (tail) mechanosensory stimulus [5, 7]: PLML/R, PVDL/R, PDEL/R
(i2) Anterior (head) mechanosensory stimulus [5, 7]: ADEL/R, ALML/R, AQR, AVM, BDUR/L, FLPL/R, SIADL/R
(i3) Posterior (tail) chemosensory stimulus (also reported as anus mechanosensory stimulus) [7, 38]: PHAL/R, PHB/R
(i4) Anterior (head) chemosensory stimulus [38]: ADLL/R, ASHL/R, ASKL/R.

**Fig 7:**
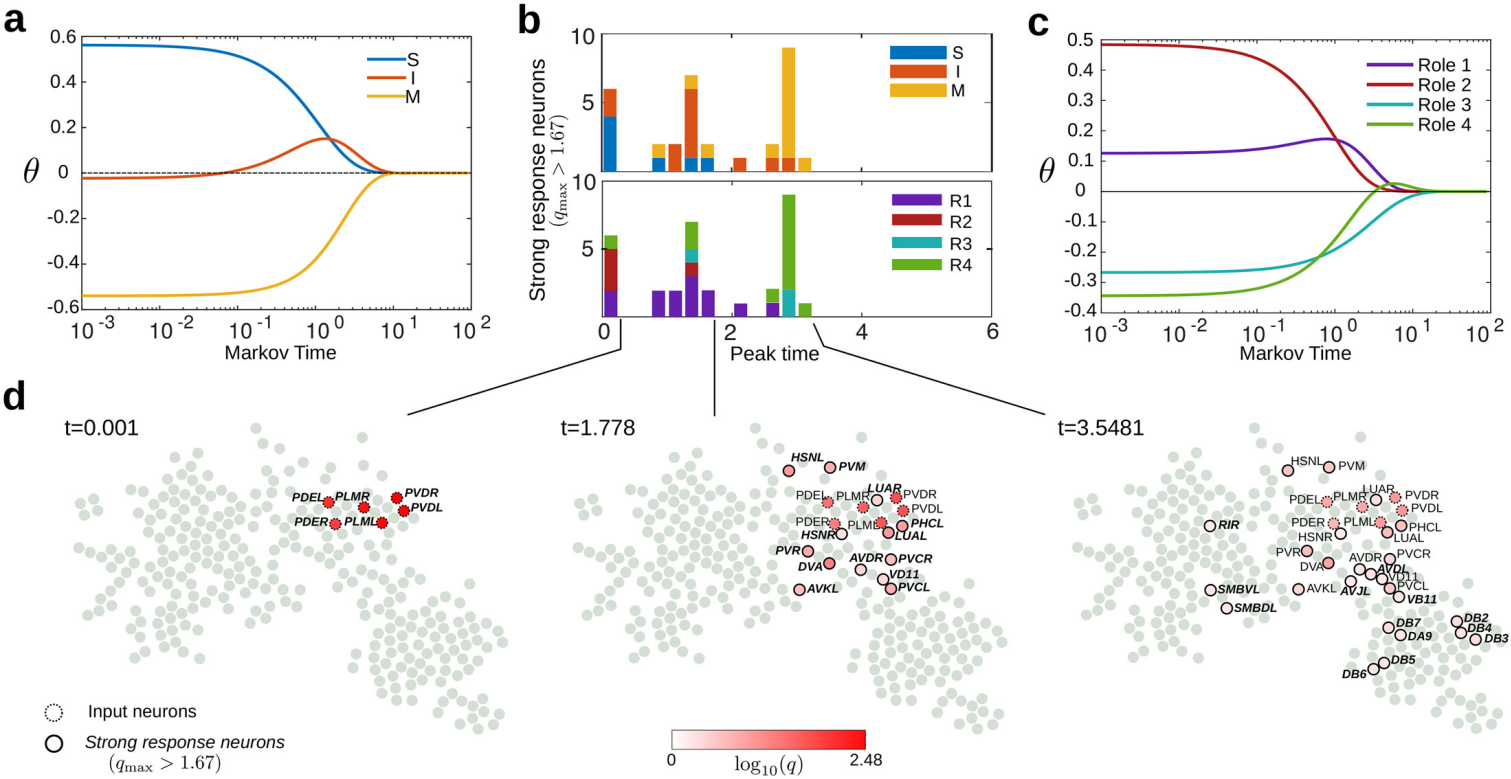
Signal propagation of posterior mechanosensory stimulus (i1). **(a)** As stationarity is approached (***θ***(*t*) → 0), the input propagates from sensory to motor neurons through an intermediate stage when interneurons overshoot. **(b)** Signal propagation as a cascade of strong response neurons (32 neurons with *q*_max,*i*_ > 1 + 2/3) with peak times concentrated around two bursts. The number of neurons are colored according to type (top) and role (bottom). Note the overall trend S → I → M during the propagation of strong responses, and how the sequence of strong response neurons also reflects the connectivity between roles propagating roughly from R2 to R1 and finally to R3. **(c)** The input (i1), which is highly localised on R2 neurons, diffuses quickly to R1 neurons and induces an overshoot of R4 neurons followed by slower diffusion into R3 neurons. **(d)** Stages of signal propagation in the network showing the strong response neurons that have peaked at each time.

We exemplify the procedure in detail through the posterior mechanosensory stimulus (i1), but detailed results for the other stimuli are provided in the S8, S9 and S10 Figs. As shown in Fig 7a, the signal proceeds ‘downstream’ following the expected biological information processing sequence, S→I→M. The signal is initially concentrated on the input neurons (mostly sensory); then propagates out primarily to interneurons, which overshoot and peak at *t* ≈ 1.5; and is then passed on to motor neurons, which slowly increase towards their stationary value.

The flow roles obtained above provide further insight into the propagation of stimuli. As seen in Fig 7c, the input for the tail mechanosensory scenario (i1) is heavily concentrated on R2 neurons (the escape response group), from which the signal flows quickly towards the other upstream (head) group R1, followed by propagation towards the downstream group R4. Finally, the signal spreads more slowly to R3, the head-centric downstream unit. This pattern of propagation carries onto the sequence of strong response neurons (Fig 7b), and reflects the fact that R2 contains posterior upstream units, and mirrors the strong connectivity of R2 with motor neurons in R1 (AVDL/R and DVA) and R4 (PVCL/R), as discussed above.

To detect key neurons comprising the specific propagation pathways, we find *strong response neurons*, i.e., those with large overshoots relative to their stationary value,

$$q_{\text{max},i}=\max_{t}q_{i}\left(t\right)>1+\frac{2}{3}.$$

See S7 Fig for a full description of the procedure. According to this criterion, we obtain 26 strong response neurons for scenario (i1). The neurons have large overshoots in two time windows after the inital input (Fig 7b). The details of the signal propagation (Fig 7d) show that a first wave of peak responses (around *t* ≈ 1) corresponds mostly to overshooting interneurons, including AVDL/R and DVA, responsible for mechanosensory integration, and PVCL/R, drivers of forward motion [5, 45]. The second wave of peaks (around *t* ≈ 3) contains predominantly ventral B-type motor neurons, e.g., DB2-7 and VB11. Such B-type motor neurons are responsible for forward motion. Hence the progression of overshooting neurons suggests a plausible biological response for a posterior mechanosensory stimulus [7, 45]. The overshooting behaviour of the neurons is not captured by other static measures of the network (e.g., in/out degree or pagerank), as shown in S12 Fig.

#### Comparison with other biological scenarios

Detailed results of propagation under the other biological scenarios (i2)-(i4) from the experimental literature are presented in S8, S9 and S10 Figs. The overall progression of the signal from S to I to M is observed with small differences in all scenarios. However, the different scenarios exhibit distinctive participation of the flow roles. In particular, both posterior stimuli (i1) and (i3) spread from R2 neurons quickly into R1 neurons and R4 (motor) neurons, with weak propagation into R3 neurons. On the other hand, anterior stimuli (i2) and (i4) spread from the R1 group strongly into R3 neurons and also quickly to R2 neurons, with only weak spreading into R4 neurons. In cases (i1)-(i3) information flows fast out of R2 towards motor neurons, as could be expected from neurons triggering an escape response. Interestingly, the (i4) scenario does not feature any strong response neurons in the R2 group.

**Fig 8:**
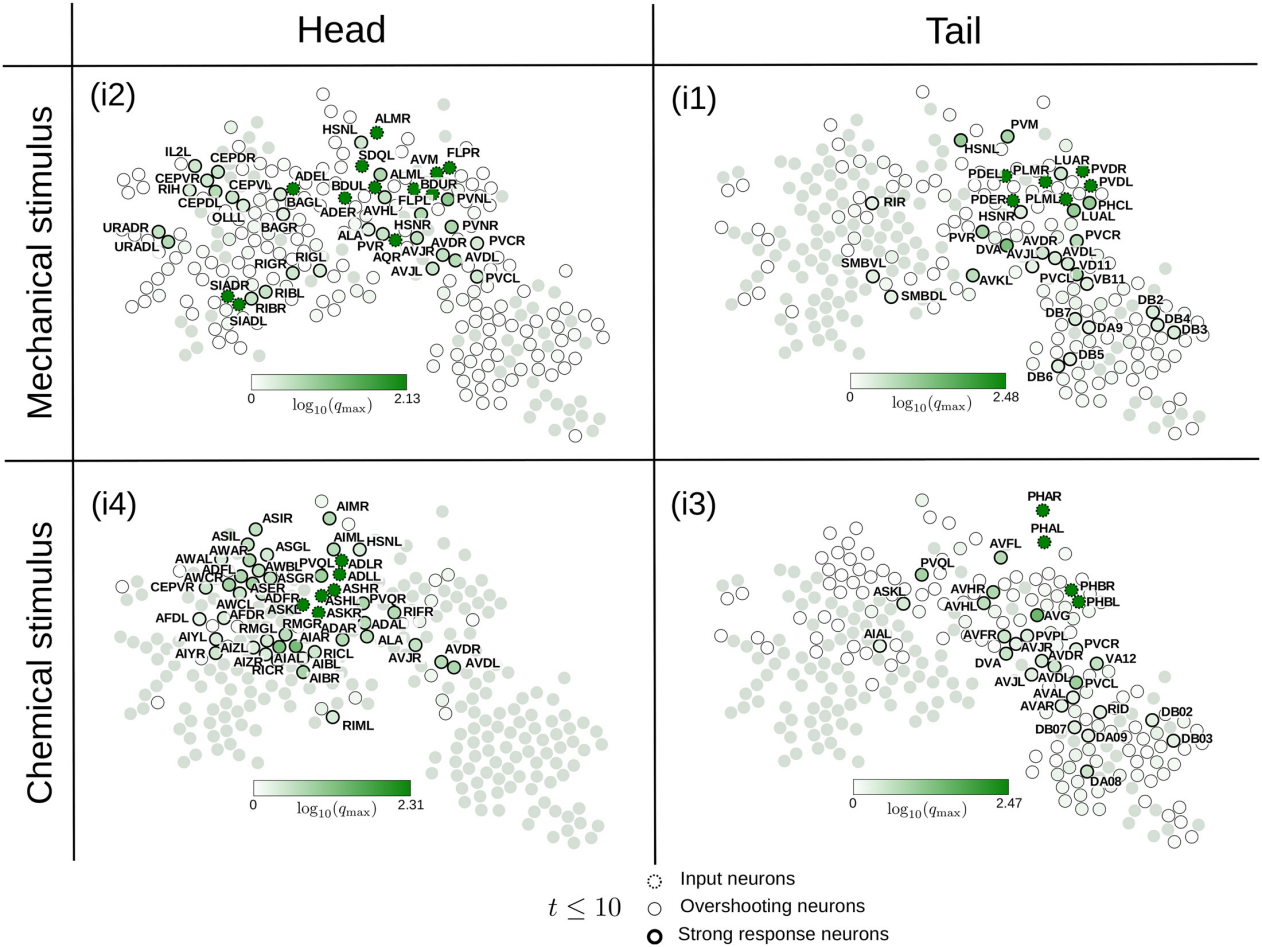
Summary of signal propagation in the four biological scenarios. The specific pathways for the signal propagation for each of the scenarios (i1)-(i4) are shown, highlighting the input, strong response (*q*_max,*i*_ > 5/3) and overshooting neurons (*q*_max,*i*_ > 1). The input and strong response neurons are labelled for each biological scenario.

As shown in S8–S10 Figs, and summarised in Fig 8, the signal propagation pathways have distinctive characteristics for each of the scenarios. For instance, although the posterior chemosensory scenario (i3) shows strong similarities to (i1) at earlier stages (input mostly R2 and strongly responding interneurons PVCL/R, AVDL/R, AVJL, DVA), they show differences in the motor neurons exhibiting a strong overshoot. In particular, for (i3) A-type neurons (DA8, DA9, VA12) responsible for backward motion are present in addition to B-type neurons (DB2, DB3, DB7).

The anterior (head) scenarios (i2) and (i4) inputs show a localised propagation mostly in head-centric groups R1 and R3. For the anterior mechanosensory scenario (i2), command interneurons such as PVCL/R, AVDL/R respond strongly, together with ring interneurons, such as RIGL/R and RIBL/R. In this case, only small excitation of ventral cord motor neurons is attained. Instead, we observe strong responses of polymodal ring motor neurons, such as URADL/R and SIADL/R, and of sensory neurons CEPVL/R and CEPDL/R, even though these CEP neurons receive no external input. Interestingly, CEP neurons are reported [45] to be functionally redundant with nose touch receptors ADE, where the input signal is located. Upon anterior chemical stimulation (i4), a bulk of flow is captured within the neuronal ring and induces strong response from chemosensory neurons such as PVQL, ASKL, AWAL/R, AWABL/R and AWACL/R, as well as interneurons RICL/R, RMGL/R, AIAL/R and AIBL/R, which are specific for integrating chemo-sensation. Indeed, several of these neurons also appear in the posterior chemosensory stimulus (i3). A summary of the strong response and overshooting neurons for all scenarios is presented in Fig 8.

## Discussion

We have presented an integrated network-theoretic analysis of the *C. elegans* connectome in terms of directed flows. We exploit the connection between diffusive processes and graph-theoretical properties, which intimately links structure and dynamics, to elucidate dynamically relevant features in the connectome. Although diffusive processes are a coarse approximation of physiological signal propagation, they can be used to extract systemic dynamical features, specifically in the case of non-spiking neuronal systems such as *C. elegans* [4].

Using the Markov Stability (MS) framework, we have identified flow-based groupings of neurons in the *C. elegans* connectome at different levels of granularity. Previous studies [20–22] have aimed at uncovering modules based on structural properties of the network, usually considering a particular scale so as to find one partition (e.g., modularity at the standard resolution). In S1 Text we provide a detailed comparison of MS multiscale flow structures against partitions found by modularity [20, 21], stochastic block models [22] and MapEquation [52]. The partitions obtained by MS at a particular scale are closer to those obtained with directed modularity. The MS framework, however, provides a multiscale description across all scales by sweeping the Markov time [33], respecting and exploiting directionality. In doing so, it reveals an intrinsic, quasi-hierarchical organisation of the connectome, giving insight into relevant features of signal propagation. The partitions found by MS are in good agreement with *C. elegans* physiology, and summarise previously observed features, such as the hierarchical and spatial organisation of neuronal communities [23, 40].

The obtained flow-based organisation highlights the prominent position of particular neurons, such as AVF and AVH, and allows for a systematic exploration of single and double ablations most disruptive of signal flows, thus providing insight into candidate neurons for further experimental investigations. Examples of such neurons include, among others: the synergistic effects caused by neuron AIAR in double ablations; the global role of D-type motor neurons, which often appear as relevant in single ablations; or the role of polymodal (I/M) SAAVL/R head neurons [45], about which little is known but which appear in the R2 group and are salient in our ablations. Several other examples are discussed in the text, and further such hypotheses may be formulated based on the full set of ablation scores we provide in S1 Data as a resource to experimentalists investigating the physiology of particular neurons.

Other methods can be used to study the effect of ablations using, for example, measures of centrality, efficiency or information transfer [53, 54]. Our study of ablations gives distinct results, as shown in S3 Fig. For instance, because our measures focus on the disruption of the flow community structure at different scales, our approach can provide a structured view of the effect of ablations for different neuron types, as shown in Figs 3–5.

As a complementary flow-based perspective, we have used Role Based Similarity to identify classes of neurons with similar patterns of flow in the *C. elegans* nervous system. Rather than reflecting any measure of connectedness in the network, such *flow roles* (or flow profiles) reflect similar roles in the generation, distribution and consumption of flow in the directed connectome. In previous work, neurons have been assigned to roles by exploring the core-periphery structure [55], or by examining the connections of nodes within and between communities [47, 56]. Other notions of roles have been based on the use of centrality scores, or on combinatorial notions of social neighbourhoods, as in regular and structural equivalence [49, 50]. RBS takes a different approach by grouping neurons according to their patterns of in/out flows at multiple scales in the graph, irrespective of their community membership and going beyond standard classifications [34, 37]. See S2 Text and S6 Fig for a comparison of RBS flow roles, regular equivalence and community roles.

The RBS analysis of flow profiles finds two groups of mostly upstream neurons and two groups of mostly downstream neurons, yet with a specific inter-connectivity pattern. In particular, the analysis singles out a small group of upstream neurons (R2), which is functionally related to escape responses from noxious factors, and could also be the object of further experimental investigation. The RBS roles are also informative in conjunction with signal propagation from ‘input-response’ *in silico* biological scenarios (see S11 Fig). In particular, the R2 group plays an important role in posterior biological stimuli, channelling stronger and faster responses, whereas R3 (the downstream, head-centric group) constitutes a self-contained set of neurons mainly accessible via the upstream, head-centric R1 group. Therefore, the propagation profiles obtained for different biological scenarios suggest a graded organisation of the roles of nodes in terms of upstream-downstream information, which could provide valuable insight into functional circuits.

Interesting theoretical extensions of the current work would include considering the *C. elegans* connectome as a multiplex network; taking into account the different types of synapse in a more explicit fashion; and enriching the dynamics of the model by incorporating the effects of inhibitory synapses and nonlinearities in the dynamics. Furthermore, one may explore more intricate dynamics by incorporating the memory of information flow using higher order Markov models [57, 58].

Our computational tools could be used in conjunction with experimental techniques, as an aid to the generation of functional hypotheses for experimental evaluation. With the eventual aim of linking wiring properties of the connectome with information processing and functional behaviour, high throughput experiments (e.g., systematic ablation of several neurons) coupled with advancements in neuronal monitoring that can allow recordings from thousands of neurons simultaneously [59] could deliver time course measurements to characterise signal propagation in relation to function. Another interesting area of future work would be the evaluation of ablation and propagation scenarios as related to quantitative behavioural investigations upon more general ablational/mutational strategies in *C. elegans* [10–12], as well as comparative studies of the flow architecture in different nematode species [60]. Such comparative analyses between the functional and structural network of the connectome could yield valuable information in bridging the relation between structure and function in network neuroscience.

## Methods

### The *C. elegans* neuronal network

The information of the large component of the connectome network is encoded into the *n* × *n* adjacency matrix *A* (*n* = 279), where entry *A*_*ij*_ counts the total number of synapses (both chemical synapses and gap junctions) connecting neuron *i* to neuron *j* [4]. Note that chemical synapses are not necessarily reciprocal, hence *A* ≠ *A*^*T*^. Therefore the connectome is a *directed, weighted network*. The network is relatively sparse, with 2990 edges: 796 edges formed by gap junctions only; 1962 containing only chemical synapses; 232 edges with both gap junctions and chemical synapses present. The vector of out-strengths, which compiles the sum of all synapses for each neuron, is **d** = *A***1** (where **1** is the *n* × 1 vector of ones). The average out-strength per neuron is 29; ranging from the maximum (256) attained by neuron AVAL to the minimum (0) attained by the motor neuron DD6, which is the only sink in the network. The network is *not* strongly connected.

### Propagation dynamics in the network

Methods with different levels of complexity have been used to study signal propagation in the *C. elegans* connectome (see, e.g., Refs. [4, 51, 61–63]). Here, we use a continuous-time diffusion process as a simple proxy for the spread of information in this neuronal network. Note that gap junctions may be simply modelled as linear resistors and, although chemical synapses are likely to introduce nonlinearities, their sigmoidal transfer functions may be well approximated by a linearisation around their operating point. Indeed, as remarked by Varshney et al. [4], such an approach has additional merit in *C. elegans*, where neurons do not fire action potentials and have chemical synapses that release neurotransmitters tonically [64]. Thus, linear systems analysis is in this case an appropriate tool that can provide valuable insights [4]. Interestingly, athough simplified, such linear models have been successfully applied even to the analysis of spatio-temporal behaviour of strongly nonlinear neuronal networks [65].

The signal on the nodes at time *t* is represented by the 1 × *n* row vector ***ϕ***(*t*) governed by the differential equation

$$\frac{d\phi }{dt}=\phi [M-I],$$ (3)

where *I* is the identity matrix and *M* is the transition matrix defined as follows:

$$M=\tau D^{†}A+\frac{1}{n} [\left(1-\tau \right)1+\tau \;1_{d_{i}=0}]1^{T}.$$ (4)

Here, *τ* ∈ (0, 1) is the Google teleportation parameter (and we take *τ* = 0.85 as is customary in the literature); $1_{d_{i}=0}$ is the indicator vector of sink nodes; and the diagonal matrix *D*^†^ is the pseudo-inverse of the degree matrix:

$$D_{ii}^{†}=\left\{\begin{array}{cc} 0 & \text{if}\;\;d_{i}=0\\ 1/d_{i} & \text{if}\;\;d_{i}\neq 0. \end{array}\right.$$

The matrix *M* describes a signal diffusion along the directed edges with an additional re-injection of external ‘environmental noise’: each node receives inputs from its neighbours (which transmit flow along their outgoing links according to their relative weight with probability *τ*) and receives a constant external re-injection of size (1 − *τ*)/*n*. For pure sinks, the outgoing flow is uniformly redistributed to all nodes so as to avoid the signal accumulating at nodes with no out-links. Mathematically, this reinjection of probability (known as teleportation in the networks literature) guarantees the existence of a unique stationary solution for Eq (3), even when the network is not strongly connected [24, 66]. Biophysically, the teleportation can be understood as modelling the random interactions with the external environment.

Let ***ϕ***(0) be the input, i.e., the signal at *t* = 0. The solution of Eq (3) is then:

$$\phi \left(t\right)=\phi \left(0\right)\exp\left(t\;[M-I]\right),$$ (5)

with stationary solution ***ϕ***(*t* → ∞) = (***ϕ***(0) ⋅ **1**)***π***, where ***π*** is the dominant left eigenvector of *M*, known as PageRank [66]. Therefore, under a unit-normalised input, ***ϕ***(*t*) ⋅ **1** = 1 ∀*t*, and the stationary solution is ***π***.

### A dynamical perspective for community detection in graphs: Markov Stability

The diffusive dynamics Eq (3) can be exploited to reveal the multiscale organisation of the *C. elegans* connectome using the Markov Stability community detection framework [24, 31, 32]. Markov Stability finds communities across scales by optimising a cost function related to this diffusion (parametrically dependent on time) over the space of all partitions.

More formally, a partition P of the *n* nodes of the network into *m* non-overlapping communities is encoded as a *n* × *m* *indicator matrix* $H_{P}$:

$${\left[H_{P}\right]}_{ic}=\left\{\begin{array}{cc} 1 & \text{if}\;\text{node}\;i\;\text{belongs}\;\text{to}\;\text{community}\;c\\ 0 & \text{otherwise.} \end{array}\right.$$ (6)

Given a partition matrix $H_{P}$, we define the time-dependent *clustered autocovariance matrix*:

$$R\left(t,H_{P}\right)={H_{P}}^{T} [\Pi \exp\left(t\left[M-I\right]\right)-\pi \pi^{T}]H_{P},$$ (7)

where *Π* = *diag*(***π***). The matrix entry ${\left[R\left(t,H_{P}\right)\right]}_{cf}$ quantifies how likely it is that a random walker starting in community *c* will end in community *f* at time *t*, minus the probability for such an event to happen by chance. To find groups of nodes where flows are trapped more strongly over time *t* than one would expect at random, we find a partition P that maximises

$$r\left(t,H_{P}\right)=\text{trace}\;R\left(t,H_{P}\right).$$ (8)

We define $r(t,H_{P})$ as the *Markov Stability* of partition P at time *t* [24, 32].

Maximising $r(t,H_{P})$ over the space of all partitions for each time *t* results in the sequence of optimal partitions:

$$P_{\text{max}}\left(t\right)=\underset{P}{arg max}\;r\left(t,H_{P}\right).$$ (9)

Although the optimisation Eq (9) is NP-hard, there exist efficient heuristic algorithms that work well in practice. In particular, it has been shown that this optimisation can be carried out using any algorithm devised for modularity maximisation [24, 31, 32]. In this work, we use the Louvain algorithm [67], which is known to offer high quality solutions whilst remaining computationally efficient. The code for Markov Stability can be found at github.com/michaelschaub/PartitionStability.

As an additional improvement of the optimisation of $P_{\text{max}}\left(t\right)$, we run the Louvain algorithm *ℓ* = 100 times with different random initialisations for each Markov time *t*, and generate an ensemble of solutions ${\left\{P_{i}\left(t\right)\right\}}_{i=1}^{\ell }$. From this ensemble, we pick the best partition $\hat{P}\left(t\right)$ according to our measure Eq (8):

$$\max_{i}\;{\left\{P_{i}\left(t\right)\right\}}_{i=1}^{\ell }\mapsto \hat{P}\left(t\right)\approx P_{\text{max}}\left(t\right).$$

Ideally, the optimised partition from the ensemble, $\hat{P}\left(t\right)$, will be close to the true optimum, $P_{\text{max}}\left(t\right)$.

To identify the important partitions across time, we use the following two robustness criteria [33, 68]:

#### Consistency of the optimised partition

A relevant partition should be a robust outcome of the optimisation, i.e., the ensemble of *ℓ* optimised solutions should be similar. To assess this consistency, we employ an information-theoretical distance between partitions: the normalised variation of information between two partitions P and $P^{\prime }$ defined as [69]:

$$VI\left(P,P^{\prime }\right)=\frac{2\Omega \left(P,P^{\prime }\right)-\Omega \left(P\right)-\Omega \left(P^{\prime }\right)}{\log(n)},$$ (10)

where $\Omega \left(P\right)=-\sum_{C}p\left(C\right)\log p\left(C\right)$ is a Shannon entropy, with p(C) given by the relative frequency of finding a node in community C in partition P; $\Omega (P,P^{\prime })$ is the Shannon entropy of the joint probability; and the factor log(*n*) ensures that the measure is normalised between [0, 1].

To quantify the robustness to the optimisation, we compute the average variation of information of the ensemble of solutions obtained from the *ℓ* Louvain runs at Markov time *t*:

$$\langle VI(t)\rangle \;=\frac{1}{\ell (\ell -1)}\sum_{i\neq j}VI\left(P_{i}\left(t\right),P_{j}\left(t\right)\right).$$ (11)

If all runs of the optimisation return very similar partitions, then 〈*VI*(*t*)〉will be small, indicating robustness of the partition to the optimisation. Hence we select partitions with low values (or dips) of 〈*VI*(*t*)〉.

#### Persistence of the partition over time

Relevant partitions should also be optimal across stretches of Markov time. Such persistence is indicated both by a plateau in the number of communities over time and a low value plateau of the cross-time variation of information:

$$VI(t,t^{\prime })\;=VI(\hat{P}\left(t\right),\hat{P}\left(t^{\prime }\right)).$$ (12)

Therefore, within a time-block of persistent partitions we choose the most robust partition, i.e., that with lowest 〈*VI*(*t*)〉.

### Quantifying the disruption of community structure under node deletion

To mimic *in silico* the ablation of neuron *i*, we remove the *i*-th row and column of the adjacency matrix *A*, and analyse the change induced in the Markov Stability community structure of the reduced (*n* − 1) × (*n* − 1) matrix *A*_[*i*]_. Double ablations are mimicked by simultaneously removing two rows (and their corresponding columns) to obtain the reduced (*n* − 2) × (*n* − 2) matrix *A*_[*i*, *j*]_.

#### Detecting salient single-node deletions

We carry out a systematic study of all single node deletions in the network. To detect relevant deletions, we monitor either an induced loss of robustness or an induced disruption in the make-up of particular partitions.

*Changes induced in the robustness of partitions.* First, we run the MS analysis on *all* deletions to obtain the optimised partitions and their robustness across all times *t*:

$${\left\{{\hat{P}}_{\left[i\right]}\left(t\right),\;\left\langle VI_{\left[i\right]}\left(t\right)\right\rangle \right\}}_{i=1}^{n}\;\forall \;t.$$ (13)

We then fit a Gaussian Process (GP) [70] to the ensemble of *n* + 1 time series of the robustness measure 〈*VI*_[*i*]_(*t*)〉, plus the unablated 〈*VI*(*t*)〉. The resulting GP, with mean *μ*(*t*) and variance *σ*^2^(*t*), describes the average robustness of partitions under a single-node deletion.

To detect single-node deletions that induce a large change in the robustness of a given partition we find sustained outliers of the GP. For a partition $\hat{P}$ optimal over *t* ∈ [*t*_1_, *t*_2_], we select node deletions *i* such that 〈*VI*_[*i*]_(*t*)〉 differs from *μ*(*t*) by at least two standard deviations *σ*(*t*) over a continuous time interval larger than $\ln(\sqrt{t_{2}/t_{1}})$ [68]. This criterion identifies node deletions that disrupt the robustness of a partition over its epoch.

*Changes induced in the make-up of partitions.* To detect if the deletion of node *i* induces a change in the make-up of partition $\hat{P}$, we compute the *community variation*:

$$CV_{\left[i\right]}\left(\hat{P}\right)=\min_{\tau }\;VI\left(\hat{P},{\hat{P}}_{\left[i\right]}\left(\tau \right)\right),$$ (14)

i.e., the variation of information between $\hat{P}$ and the most similar among *all* optimal partitions of the ablated network ${\hat{P}}_{\left[i\right]}\left(t\right)$.

We detect outliers in *CV* for each partition using a simple criterion based on the inter-percentile range: the deletion of *i* is considered an outlier if *CV*_[*i*]_ > *P*_90_ + *IPR*_90/10_, where *P*_90_ is the 90th percentile, and *IPR*_90/10_ = |*P*_90_ − *P*_10_| is the interpercentile range between the 10th-90th percentiles of the ensemble of *CV*_[*i*]_.

#### Detecting supra-additive double-node deletions

We have carried out a study of all double deletions in the network to detect two-node deletions whose effect is larger than the additive effect of the two corresponding single node deletions. To this end, we first obtain the set of MS partitions across all Markov times for all double delections ${\hat{P}}_{\left[i,j\right]}\left(t\right)$, and compute their community variation:

$$CV_{\left[i,j\right]}\left(\hat{P}\right)=\min_{\tau }\;VI\left(\hat{P},{\hat{P}}_{\left[i,j\right]}\left(\tau \right)\right).$$ (15)

We then compute the average of the individual ablations:

$$CV_{\left[i],[j\right]}\left(\hat{P}\right)=\frac{CV_{\left[i\right]}\left(\hat{P}\right)+CV_{\left[j\right]}\left(\hat{P}\right)}{2}.$$ (16)

To find pairs with a supra-additive effect, we use Quantile Regression (QR) [71], a method widely used in econometrics, ecology, and medical statistics. Whereas least squares regression aims to estimate the conditional mean of the samples, QR provides a method to estimate conditional quantiles of the sample distribution. Hence, QR facilitates a more global representation of the relationships between the dependent and independent variables considered in the regression. A good introduction to QR can be found in Ref. [72], and a more in-depth treatment can be found in the book by Koenker [71].

For a partition $\hat{P}$, we employ QR to fit quantiles for the regression of $CV_{\left[i,j\right]}\left(\hat{P}\right)$ against $CV_{\left[i],[j\right]}\left(\hat{P}\right)$, using all 38781 two-node ablations (Fig 5). We report the top 1% double deletions according to their quantile scores—this is our criterion to select double-ablations that have a strong effect. All scores are computed using Bayesian Quantile Regression, as implemented in the R package BSquare (https://cran.r-project.org/web/packages/BSquare/index.html), which fits all quantiles simultaneously resulting in a more coherent estimate [73]. Following Ref. [73], we fit the quantiles to the normalised $CV_{\left[i],[j\right]}\left(\hat{P}\right)$ using a Gamma centering distribution and four basis functions.

### Finding flow roles in networks: Role-based similarity

In directed networks, nodes can have different ‘roles’, e.g., sinks, sources or hubs. In complex directed networks, functional roles may not fall into such simple categories, yet nodes can still be characterised by their contribution to the diffusion of in- and out-flows. Here we use a recent method (Role-Based Similarity, RBS) to uncover roles in directed networks based on the patterns of incoming and outgoing flows at all scales [35, 36]. The main idea underpinning RBS is that nodes with a similar in/out flow profile play a similar role, regardless of whether they are near or far apart in the network. Each node is associated with a feature vector **x**_*i*_ containing a weighted number of in- and out-paths of increasing lengths beginning and ending at the node. The feature vectors are collected in the feature matrix *X*:

$$X= [\begin{array}{c} x_{1}\\ \vdots \\ x_{n} \end{array}]=\overset{\text{paths}\;\text{in}}{\overset{︷}{ [\cdots {\left(\beta A^{T}\right)}^{k}1\cdots }}|\overset{\text{paths}\;\text{out}}{\overset{︷}{\left.\cdots {\left(\beta A\right)}^{k}1\cdots \right]}},$$ (17)

where *β* = *α*/*λ*_1_, with *λ*_1_ the spectral radius of the adjacency matrix *A* and *α* ∈ (0, 1). The cosine between feature vectors gives the similarity score between nodes:

$$Y_{ij}=\frac{x_{i}x_{j}^{T}}{{\|x_{i}\|}_{2}\;{\|x_{j}\|}_{2}}.$$ (18)

The *n* × *n* matrix *Y* quantifies how similar the directed flow profiles between every pair of nodes are. Nodes with identical connectivity have *Y*_*ij*_ = 1, whereas in the case of nodes with dissimilar flow profiles (e.g., if *i* is a source node with no incoming connections and *j* is a sink node with no outgoing connections), then their feature vectors are orthogonal and *Y*_*ij*_ = 0.

As outlined in Refs. [35–37], we compute the similarity matrix *Y* iteratively with *α* = 0.95, and apply the RMST algorithm to obtain a *similarity graph*, in which only the important information of *Y* is retained. We then extract *flow roles* in a data-driven manner without imposing the number of roles *a priori* by clustering the similarity graph (see S4 Fig). The flow roles so obtained have been shown to capture relevant features in complex networks, where other role classifications based on combinatorial concepts and neighbourhoods fail [34, 37]. In particular, our flow roles are fundamentally different from notions of roles in social networks based on Structural Equivalence [49] and Regular Equivalence [50]. Such equivalence measures do not incorporate information about the large scales of the network and are sensitive to small perturbations, making them unsuitable for complex networks such as the *C. elegans* connectome [34] (see S6 Fig for roles based on Regular Equivalence).

## Supporting Information

S1 Data Supplementary Data as XLS spreadsheet. (XLSX)

S1 Text Comparison of MS partitions to other methods. (PDF)

S2 Text Comparison of RBS flow roles with other analyses of roles. (PDF)

S1 FigFull analysis of the *C. elegans* connectome with Markov Stability (MS).We show the scan across all Markov times, from the finest possible partition (every node in its own partition) at small Markov times to the bipartition at large Markov times. The highlighted time interval corresponds to Fig 1 in the main text, which focusses on the medium to coarse partitions A-E.(TIF)

S2 FigThe asymmetry in the normalised conditional entropy of the optimised MS partitions signals a quasi-hierarchical community structure.The normalized conditional entropy $\Omega \left(P\left(t^{\prime }\right)|P\left(t\right)\right)/\log\left(n\right)\in \left[0,1\right]$ quantifies the uncertainty in the community assignment $P(t^{\prime })$ given the known partition P(t). If $P(t^{\prime })$ can be predicted from P(t), (i.e. when $P(t^{\prime })$ is a strictly hierarchical agglomeration of the communities of P(t)) then the conditional entropy will be zero. The strong upper-triangular character of the conditional entropy of the partitions A-E indicates a quasi-hierarchical organisation.(TIF)

S3 FigThe effect of ablations and other network measures.Scatter plots of the Community Variation with respect to Partitions A and D, $CV_{\left[i\right]}\left(A\right)$ (left column) and $CV_{\left[i\right]}\left(D\right)$ (right column), for all single neuron ablations (*i* = 1, …, 279) plotted against the following properties of the corresponding neuron: **a**, stationary flow distribution *π* (PageRank); **b**, in-degree; **c**, out-degree; **d**, betweenness centrality; and **e**, local clustering coefficient. None of these quantities (which are related to network centralities) shows a manifest correlation with the effect of the neuron ablation on community structure.(TIF)

S4 Fig Finding role profiles with RBS. Schematic summary of the procedure to obtain flow roles using RBS analysis, as discussed in detail in [37]. First, from the original *directed* network of the *C. elegans* connectome we create a similarity matrix using the RBS metric, by computing a similarity score between each node in the network, based on their incoming and outgoing weighted path profiles. Second, the similarity matrix is transformed into a similarity matrix using the RMST method, which subsequently prunes out uninformative links (see Ref. [37] for details). Third, the resulting similarity graph is clustered to obtain relevant groups of nodes with similar in- and out-flow profiles at all scales. Four such classes of neurons (*flow roles*) are found in this case. The neurons are then colored according to their flow profile on the original connectome layout.
(TIF)

S5 FigDistribution of RBS flow roles across MS communities.RBS roles in each of the six communities of partition A. The communities and flow roles induce very different groupings in the connectome. Hence the six communities present distinct mixes of roles: the anterior communities A1 and A2 present a dominance of roles R1 and R3, whereas the posterior communities A3, A4 and A5 are dominated by roles R1 and R4. Community A6 has a balanced mix of roles R1, R2, and R4 giving it a distinctive information processing structure, confirming the the importance of its embedded rich-club neurons.(TIF)

S6 Fig Comparison of RBS flow roles to roles obtained using Regular Equivalence. **a**: Roles of the nodes according to RBS with the PageRank distribution for each role and the average in/out degree for each role. **b**: Same for the roles obtained according to Regular Equivalence obtained using the REGE algorithm [74].
(TIF)

S7 Fig Summary of the procedure for signal propagation analysis of posterior mechanosensory stimulus scenario (i1). For all neurons, we compute *ϕ*_*i*_(*t*), i.e., the amount of signal present at each node at Markov time *t*. As time grows, the signal at each node converges to its stationary value *π*_*i*_. Hence *θ*_*i*_(*t*) = *ϕ*_*i*_(*t*) − *π*_*i*_ → 0. The approach to stationarity can happen in two ways: i) the initially negative *θ*_*i*_(*t*) approaches 0 from below; ii) *θ*_*i*_(*t*) ‘overshoots’ before decaying towards its stationary value. We consider the signal relative to the stationary value, *q*_*i*_(*t*) = *ϕ*_*i*_(*t*)/*π*_*i*_, and focus on neurons that overshoot (i.e., those with *q*_max, *i*_: = max_*t*_ *ϕ*_*i*_(*t*)/*π*_*i*_ > 1) and we collect the times at which they reach their peak. A concise summary of the signal propagation is given by the *strong response neurons* with *q*_max,*i*_ > 5/3. Their peak-time histogram and the particular sequence of strong response neurons is characteristic of the different input-response biological scenarios, as well as the analyses by neuron type and flow roles.
(TIF)

S8 Fig Signal propagation of the anterior mechanosensory stimulus (i2). Signal propagation evolving from an initial condition localised at the mechanosensory neurons (i2). **(a)** As stationarity is approached (***θ***(*t*) → 0), the input propagates from sensory to motor neurons through an intermediate stage when interneurons overshoot. **(b)** The propagation seen as a cascade of strong response neurons (*q*_max,*i*_ > 1 + 2/3) with peak times concentrated around two bursts. **(c)** The input (i2), appears localised on R1 and to a lesser extent R2 neurons. The signal diffuses somewhat quicker out of R2 than R1 neurons, but induces not collective overshoot of R3 or R4 neurons. **(d)** Stages of signal propagation in the network showing the strong response neurons that have peaked at each time.
(TIF)

S9 Fig Signal propagation: posterior chemosensory stimulus (i3). See caption of S8 Fig.
(TIF)

S10 Fig Signal propagation: anterior chemosensory stimulus (i4). See caption of S8 Fig.
(TIF)

S11 Fig Peak times of strong response neurons by RBS roles for each of the four input scenarios (i1)-(i4). Histograms of peak times of the strong response neurons in the four biological scenarios from the perspective of flow roles. The tail inputs (i1) and (i3) induce strong responses on neurons spreading from R2 to R1 and finally to R4. On the other hand, the head inputs induce strong responses on neurons heavily based on R1 spreading downwards to R3.
(TIF)

S12 Fig Peak overshoots against other network measures. The maximum overshoot of each neuron *q*_max, *i*_ for each of the four biological scenarios (i1)–(i4) is plotted against the following measures of the corresponding neuron: **a**, stationary flow distribution ***π*** (PageRank); **b**, in-degree; **c**, out-degree; **d**, betweenness centrality; and **e**, local clustering coefficient. There is no manifest correlation between the overshooting *q*_max, *i*_ and any of those centrality scores or the local clustering coefficient.
(TIF)
