# Supplementary material for: Flow-Based Network Analysis of the Caenorhabditis elegans Connectome
Source: PLoS Comput Biol. 2016 Aug 5;12(8):e1005055. doi: 10.1371/journal.pcbi.1005055 (PMC4975510; doi:10.1371/journal.pcbi.1005055)
Supplement: S1 Text — (PDF) [file pcbi.1005055.s002.pdf]

Flow-based network analysis of the *Caenorhabditis elegans* connectome - S1 Text

Karol A. Bacik,[1,*] Michael T. Schaub,[1,2,3,†] Mariano Beguerisse-Díaz,[1,4,‡] Yazan N. Billeh,[5] and Mauricio Barahona[1,§]

[1] *Department of Mathematics, Imperial College London, London SW7 2AZ, United Kingdom*
[2] *naXys & Department of Mathematics, University of Namur, B-5000 Namur, Belgium*
[3] *ICTEAM, Université catholique de Louvain, B-1348 Louvain-la-Neuve, Belgium*
[4] *Mathematical Institute, University of Oxford, Oxford OX2 6GG, United Kingdom*
[5] *Computation and Neural Systems Program, California Institute of Technology, CA 91125 Pasadena, USA*

(Dated: June 7, 2016)

COMPARISON OF MS PARTITIONS TO OTHER METHODS:

The flow-based MS partitions are distinct from partitions obtained by several other methods. In particular, we have compared against partitions obtained with Modularity, Stochastic Block models, and Infomap.

Modularity has been used to obtain optimised partitions in Refs. [1, 2]. The partition found in Ref. [1] is closest to our 4-way Partition $\mathcal{B}$ ($VI = 0.185$), whereas the partition found in Ref. [2] is closest to our 3-way Partition $\mathcal{C}$ ($VI = 0.186$). Note that optimisation of modularity at a fixed resolution imposes an intrinsic scale, so that partitions found with modularity are well matched to a particular scale (i.e., a particular Markov time) in the Markov Stability framework, as shown previously [3, 4]. On the other hand, as discussed in the main text, the Markov Stability framework carries out a systematic scanning across Markov times [5] allowing the intrinsic multiscale organisation to became apparent.

The partitions based on stochastic block models [6] and hierarchical Infomap [7] are less similar to the ones found by MS: the partition found by stochastic block models in [6] is closest to our 3-way Partition $\mathcal{C}$ (but with a higher VI=0.272), and the partition found by hierarchical Infomap in [7] is closest to our 6-way Partition $\mathcal{A}$ (yet with an even higher VI=0.282). These differences in the outcomes are expected due to the contrasting methodological approaches. In particular, Infomap is known to impose a clique-like structure to the modules leading to groupings where strong local density is favoured [8]. We remark that, as shown in S2 Text, the MS communities are also different from the flow roles found through RBS.

---

[1] R. K. Pan, N. Chatterjee, and S. Sinha, PLOS ONE **5** (2010).
[2] Y. Sohn, M.-K. Choi, Y.-Y. Ahn, J. Lee, and J. Jeong, PLoS Comput. Biol. **7** (2011).
[3] J.-C. Delvenne, S. N. Yaliraki, and M. Barahona, Proceedings of the National Academy of Sciences **107**, 12755 (2010), arXiv:0812.1811.
[4] R. Lambiotte, J. Delvenne, and M. Barahona, Network Science and Engineering, IEEE Transactions on **1**, 76 (2014), see also arXiv:0812.1770.
[5] M. T. Schaub, J.-C. Delvenne, S. N. Yaliraki, and M. Barahona, PLoS ONE **7**, e32210 (2012).
[6] D. M. Pavlovic, P. E. Vertes, E. T. Bullmore, W. R. Schafer, and T. E. Nichols, PLoS ONE **9(7)** (2014).
[7] D. Edler and M. Rosvall, "The MapEquation software package, available online at *http://www.mapequation.org*,".
[8] M. T. Schaub, R. Lambiotte, and M. Barahona, Phys. Rev. E **86**, 026112 (2012).

* karol.bacik13@imperial.ac.uk
† michael.schaub@uclouvain.be
‡ beguerisse@maths.ox.ac.uk
§ m.barahona@imperial.ac.uk
