# Supplementary material for: Flow-Based Network Analysis of the Caenorhabditis elegans Connectome
Source: PLoS Comput Biol. 2016 Aug 5;12(8):e1005055. doi: 10.1371/journal.pcbi.1005055 (PMC4975510; doi:10.1371/journal.pcbi.1005055)
Supplement: S2 Text — (PDF) [file pcbi.1005055.s003.pdf]

Flow-based network analysis of the *Caenorhabditis elegans* connectome - S2 Text

Karol A. Bacik,[1,*] Michael T. Schaub,[1,2,3,†] Mariano Beguerisse-Díaz,[1,4,‡] Yazan N. Billeh,[5] and Mauricio Barahona[1,§]

[1] *Department of Mathematics, Imperial College London, London SW7 2AZ, United Kingdom*
[2] *naXys & Department of Mathematics, University of Namur, B-5000 Namur, Belgium*
[3] *ICTEAM, Université catholique de Louvain, B-1348 Louvain-la-Neuve, Belgium*
[4] *Mathematical Institute, University of Oxford, Oxford OX2 6GG, United Kingdom*
[5] *Computation and Neural Systems Program, California Institute of Technology, CA 91125 Pasadena, USA*

(Dated: June 7, 2016)

COMPARISON OF RBS FLOW ROLES WITH OTHER ANALYSES OF ROLES

Our RBS *flow roles* are fundamentally different from notions of roles used in social networks based on Structural Equivalence (SE) [1], and Regular Equivalence (RE) [2] (Fig. S6 and S1 Data). Because both RE and SE consider only one-step neighbourhoods and do not incorporate information about the long scales of the network [3], they are less applicable to complex networks such as the *C. elegans* connectome [4]. In particular the roles produced by REGE show undifferentiated PageRank and connectivity profiles.

In Refs. [5, 6], roles were assigned to neurons according to 'community roles', the technique proposed by Guimera et al [7] which identifies certain interneurons as relevant hubs between predefined communities. It was found that command interneurons (e.g. AVA, AVB, AVD, PVC) play the role of global hubs, whereas D-type motor neurons play the role of provincial hubs [5]. These features are in line with our ablation results, where D-type motor neuron ablations alter flows at finer scales and ablation of interneurons modifies flow patterns at larger scales. Indeed, the concept of 'community role' is closer to our ablation results, in that we measure there the disruption of flow-based communities.

Chatterjee and Sinha [8] explored the core-periphery structure of the *C. elegans* connectome using a k-core decomposition based on in- and out- degree separately. The k-core of a network is the subgraph with the property that all nodes have (in/out)degree at least k. As expected, motor neurons are overrepresented in the k-cores based on in-degree, and sensory neurons are overrepresented in k-cores based on out-degree. This distinction between neurons with upstream and downstream roles is also an inherent characteristic in the RBS analysis, yet from a different perspective, i.e., based on the global characteristics of a node with respect to the in- and out-flows in the network, rather than based on its local connections.

To quantify the differences between the groupings into roles obtained by these different methods, we have computed the variation of information between them. RBS roles show very low similarity to any of the other groupings with values of $VI = 0.3061, 0.3607, 0.4356$ against REGE [9], Sohn et al. [6] and Pan et al. [5], respectively.

[1] F. Lorrain and H. C. White, The Journal of Mathematical Sociology **1**, 49 (1971).
[2] M. G. Everett and S. P. Borgatti, The Journal of Mathematical Sociology **19**, 29 (1994).
[3] M. T. Schaub, J.-C. Delvenne, S. N. Yaliraki, and M. Barahona, PLoS ONE **7**, e32210 (2012).
[4] M. Beguerisse-Díaz, G. Garduño Hernández, B. Vangelov, S. N. Yaliraki, and M. Barahona, J R Soc Interface **11** (2014), 10.1098/rsif.2014.0940.
[5] R. K. Pan, N. Chatterjee, and S. Sinha, PLOS ONE **5** (2010).
[6] Y. Sohn, M.-K. Choi, Y.-Y. Ahn, J. Lee, and J. Jeong, PLoS Comput. Biol. **7** (2011).
[7] R. Guimera and L. A. N. Amaral, Nature **433**, 895 (2005).
[8] N. Chatterjee and S. Sinha, "Understanding the mind of a worm: hierarchical network structure underlying nervous system function in C. elegans," in *Models of Brain and Mind - Physical, Computational and Psychological Approaches*, Progress in Brain Research, Vol. 168 (Elsevier, 2007) pp. 145–153.
[9] S. P. Borgatti and M. G. Everett, Social Networks **15**, 361 (1993).

* karol.bacik13@imperial.ac.uk
† michael.schaub@uclouvain.be
‡ beguerisse@maths.ox.ac.uk
§ m.barahona@imperial.ac.uk
