# Supplementary figures and images for: Flow-Based Network Analysis of the Caenorhabditis elegans Connectome
Source: PLoS Comput Biol. 2016 Aug 5;12(8):e1005055. doi: 10.1371/journal.pcbi.1005055 (PMC4975510; doi:10.1371/journal.pcbi.1005055)

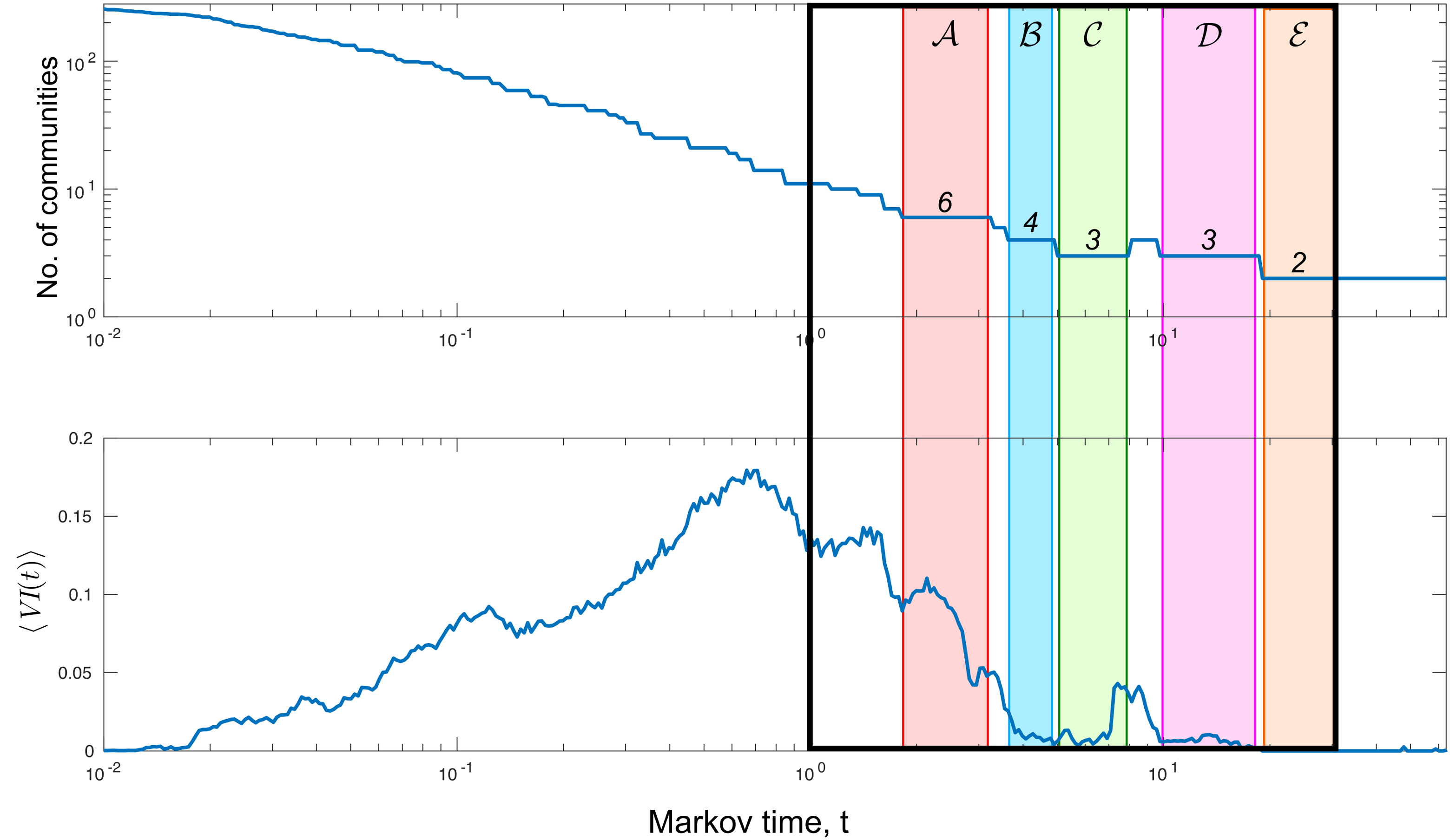

Supplement: S1 Fig — We show the scan across all Markov times, from the finest possible partition (every node in its own partition) at small Markov times to the bipartition at large Markov times. The highlighted time interval corresponds to Fig 1 in the main text, which focusses on the medium to coarse partitions A-E. (TIF) [file pcbi.1005055.s004.tif]

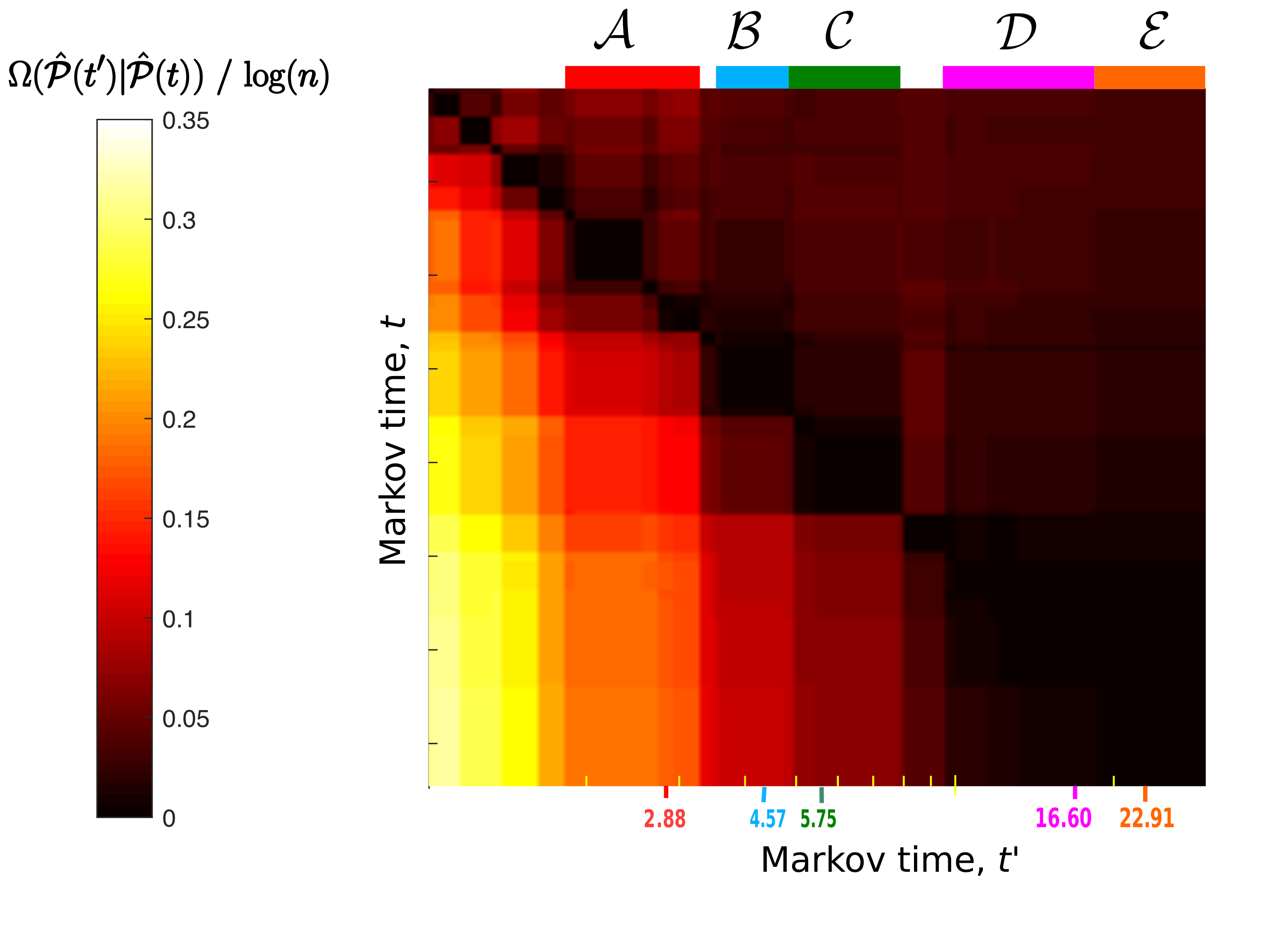

Supplement: S2 Fig — The normalized conditional entropy Ω(P(t′)|P(t))/log(n)∈[0,1] quantifies the uncertainty in the community assignment P(t′) given the known partition P(t). If P(t′) can be predicted from P(t), (i.e. when P(t′) is a strictly hierarchical agglomeration of the communities of P(t)) then the conditional entropy will be zero. The strong upper-triangular character of the conditional entropy of the partitions A-E indicates a quasi-hierarchical organisation. (TIF) [file pcbi.1005055.s005.tif]

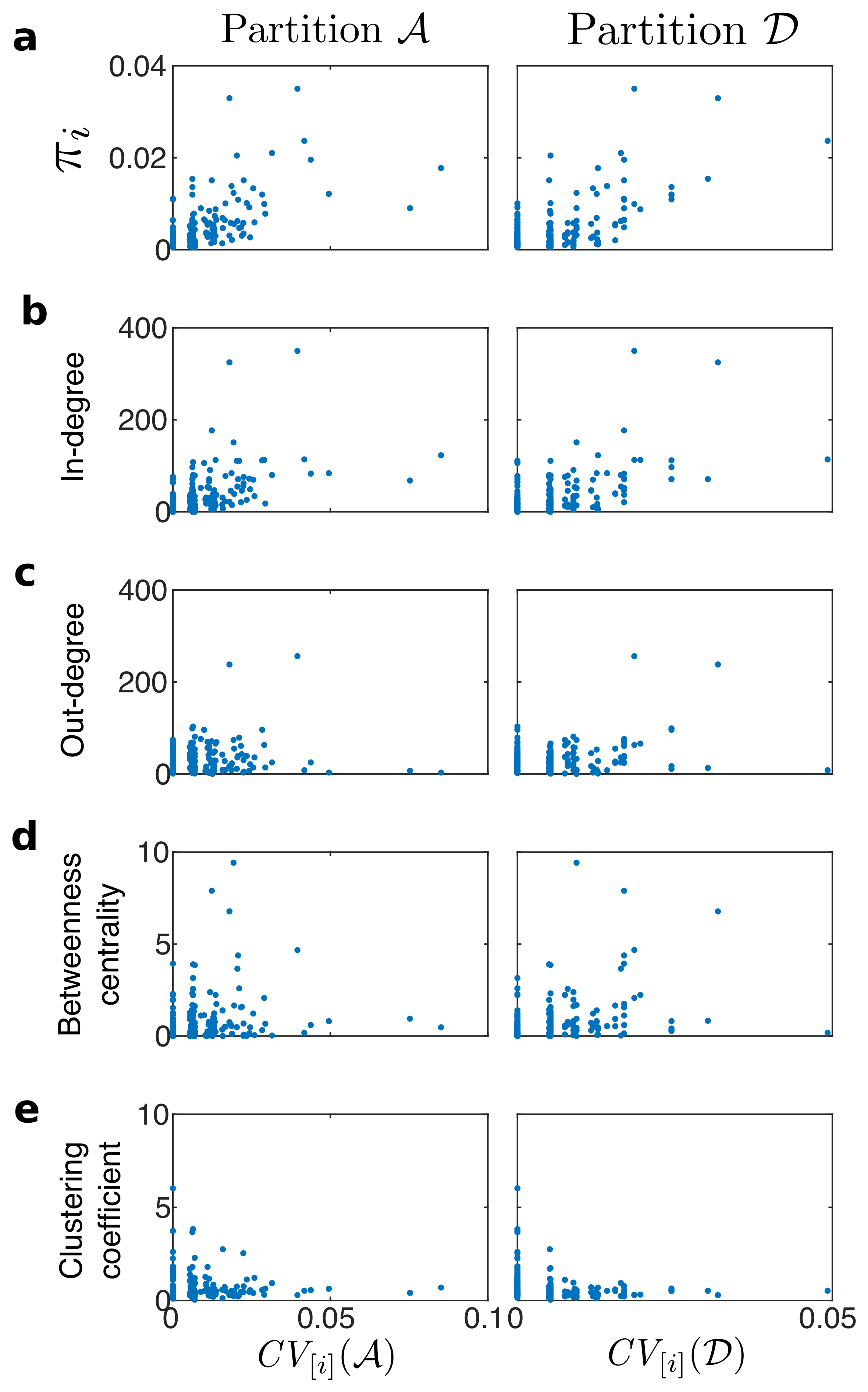

Supplement: S3 Fig — Scatter plots of the Community Variation with respect to Partitions A and D, CV[i](A) (left column) and CV[i](D) (right column), for all single neuron ablations (i = 1, …, 279) plotted against the following properties of the corresponding neuron: a, stationary flow distribution π (PageRank); b, in-degree; c, out-degree; d, betweenness centrality; and e, local clustering coefficient. None of these quantities (which are related to network centralities) shows a manifest correlation with the effect of the neuron ablation on community structure. (TIF) [file pcbi.1005055.s006.tif]

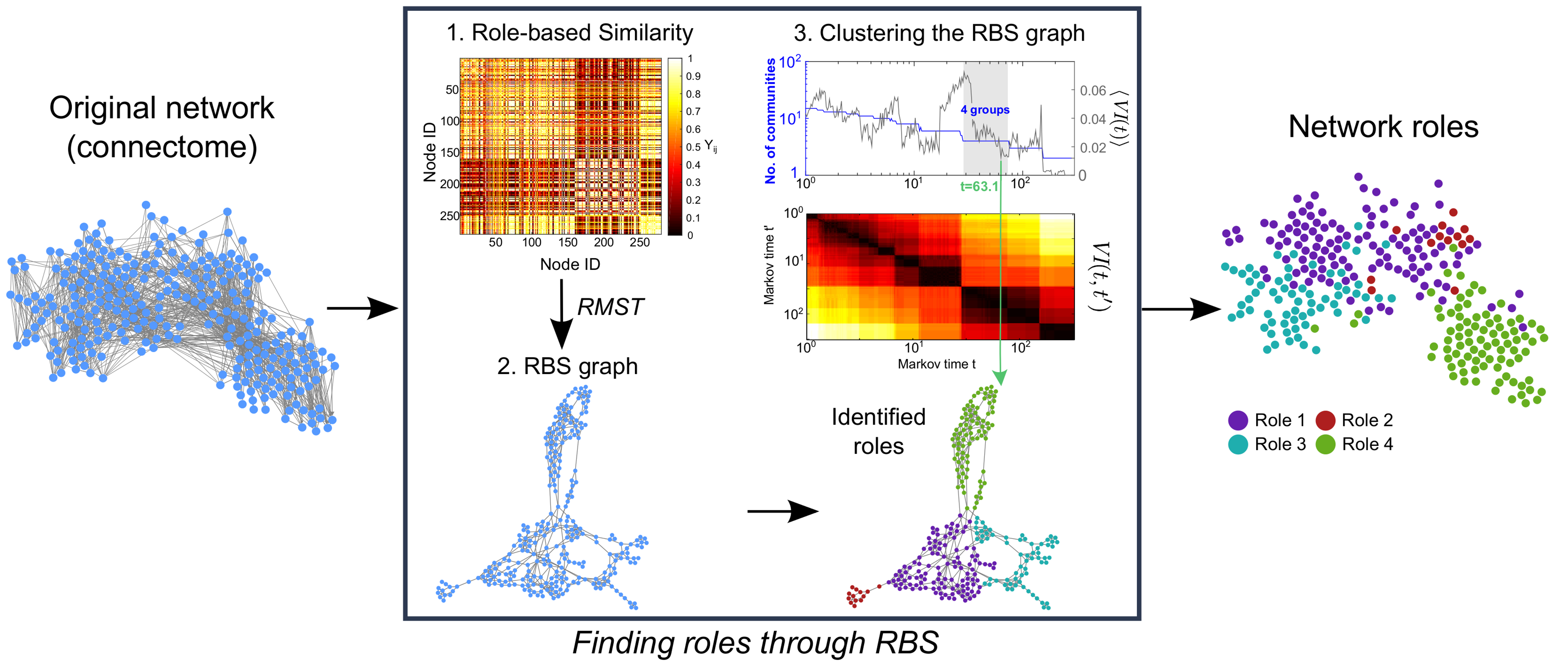

Supplement: S4 Fig — Schematic summary of the procedure to obtain flow roles using RBS analysis, as discussed in detail in [37]. First, from the original directed network of the C. elegans connectome we create a similarity matrix using the RBS metric, by computing a similarity score between each node in the network, based on their incoming and outgoing weighted path profiles. Second, the similarity matrix is transformed into a similarity matrix using the RMST method, which subsequently prunes out uninformative links (see Ref. [37] for details). Third, the resulting similarity graph is clustered to obtain relevant groups of nodes with similar in- and out-flow profiles at all scales. Four such classes of neurons (flow roles) are found in this case. The neurons are then colored according to their flow profile on the original connectome layout. (TIF) [file pcbi.1005055.s007.tif]

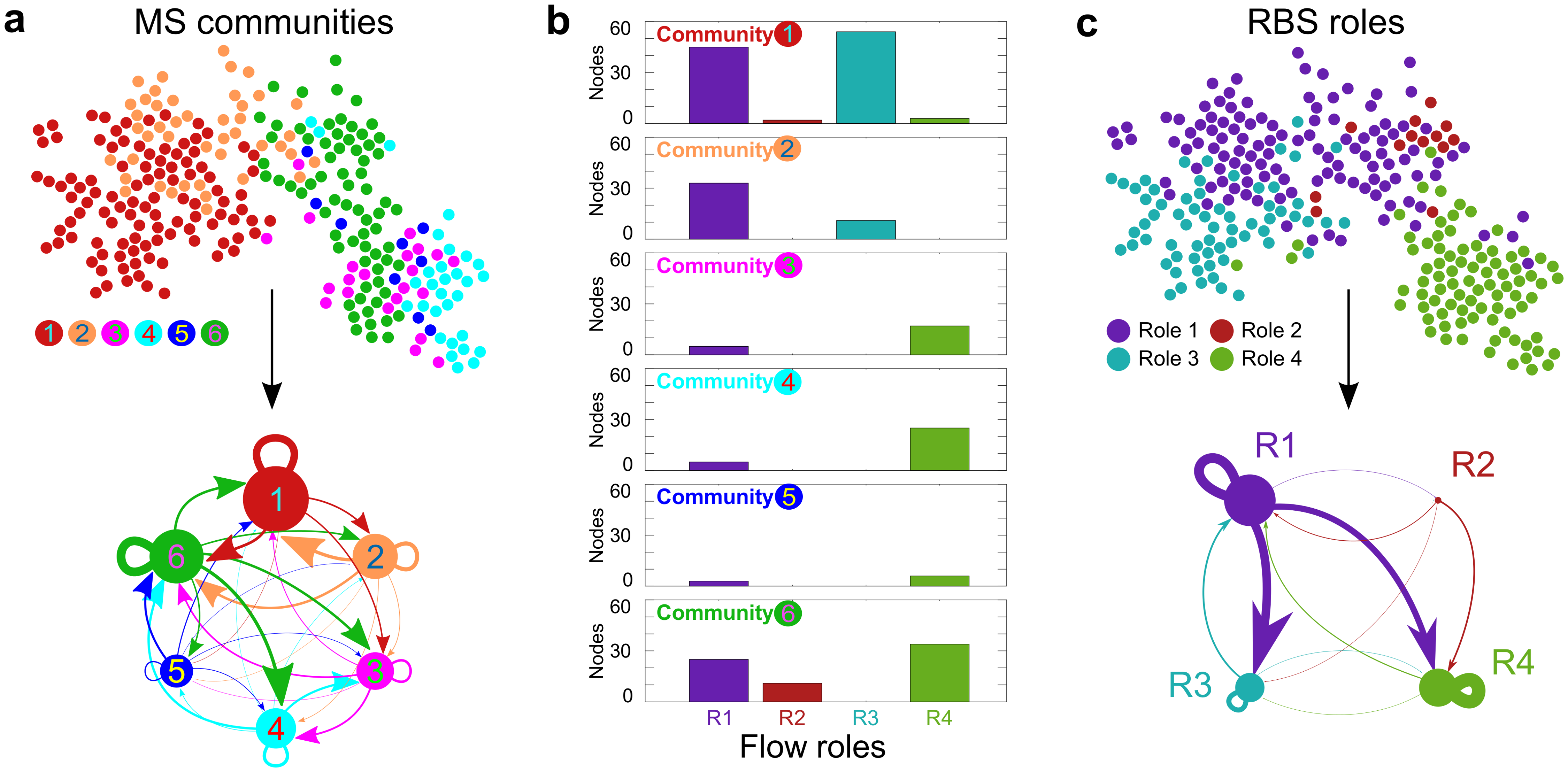

Supplement: S5 Fig — RBS roles in each of the six communities of partition A. The communities and flow roles induce very different groupings in the connectome. Hence the six communities present distinct mixes of roles: the anterior communities A1 and A2 present a dominance of roles R1 and R3, whereas the posterior communities A3, A4 and A5 are dominated by roles R1 and R4. Community A6 has a balanced mix of roles R1, R2, and R4 giving it a distinctive information processing structure, confirming the the importance of its embedded rich-club neurons. (TIF) [file pcbi.1005055.s008.tif]

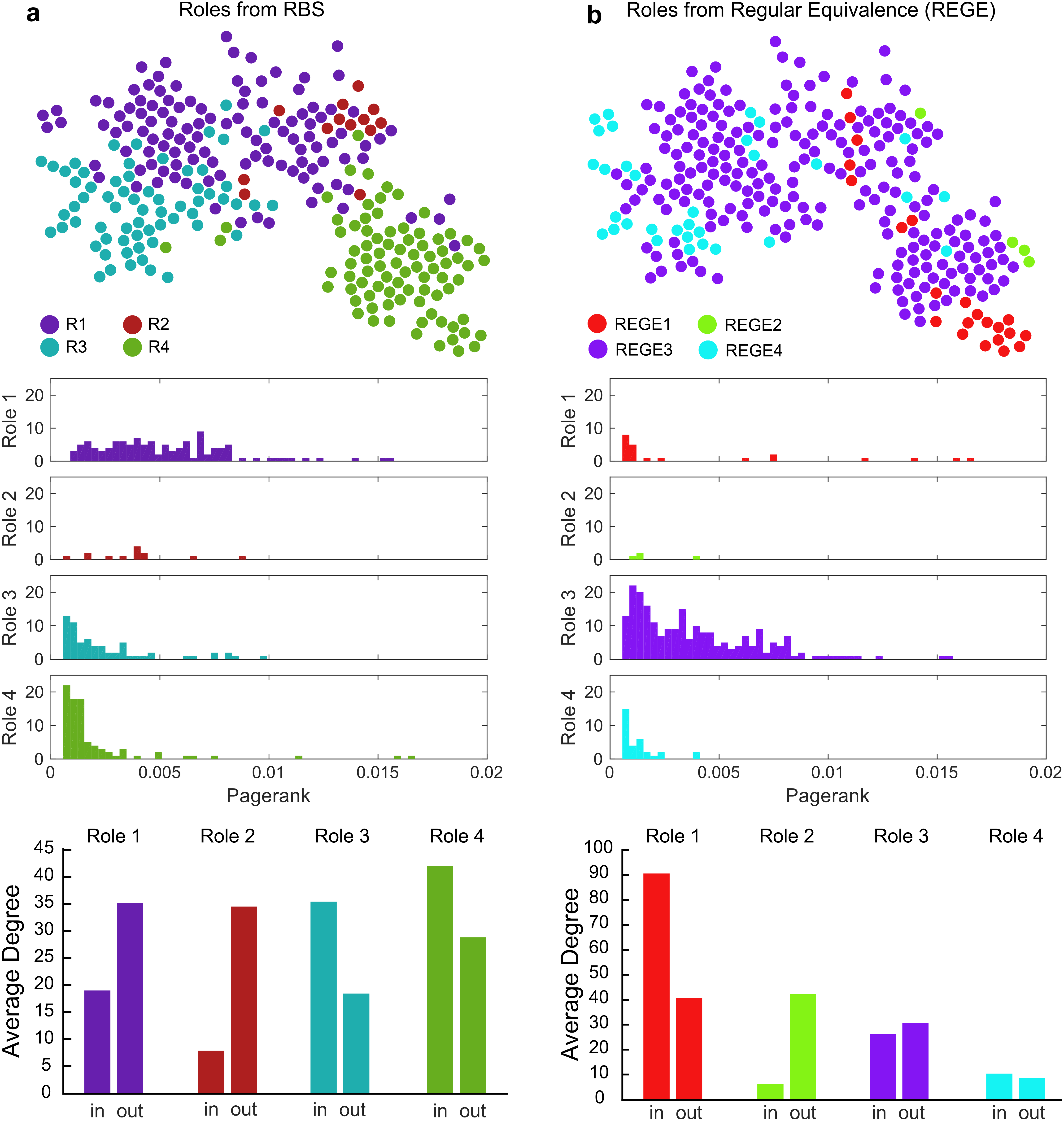

Supplement: S6 Fig — a: Roles of the nodes according to RBS with the PageRank distribution for each role and the average in/out degree for each role. b: Same for the roles obtained according to Regular Equivalence obtained using the REGE algorithm [74]. (TIF) [file pcbi.1005055.s009.tif]

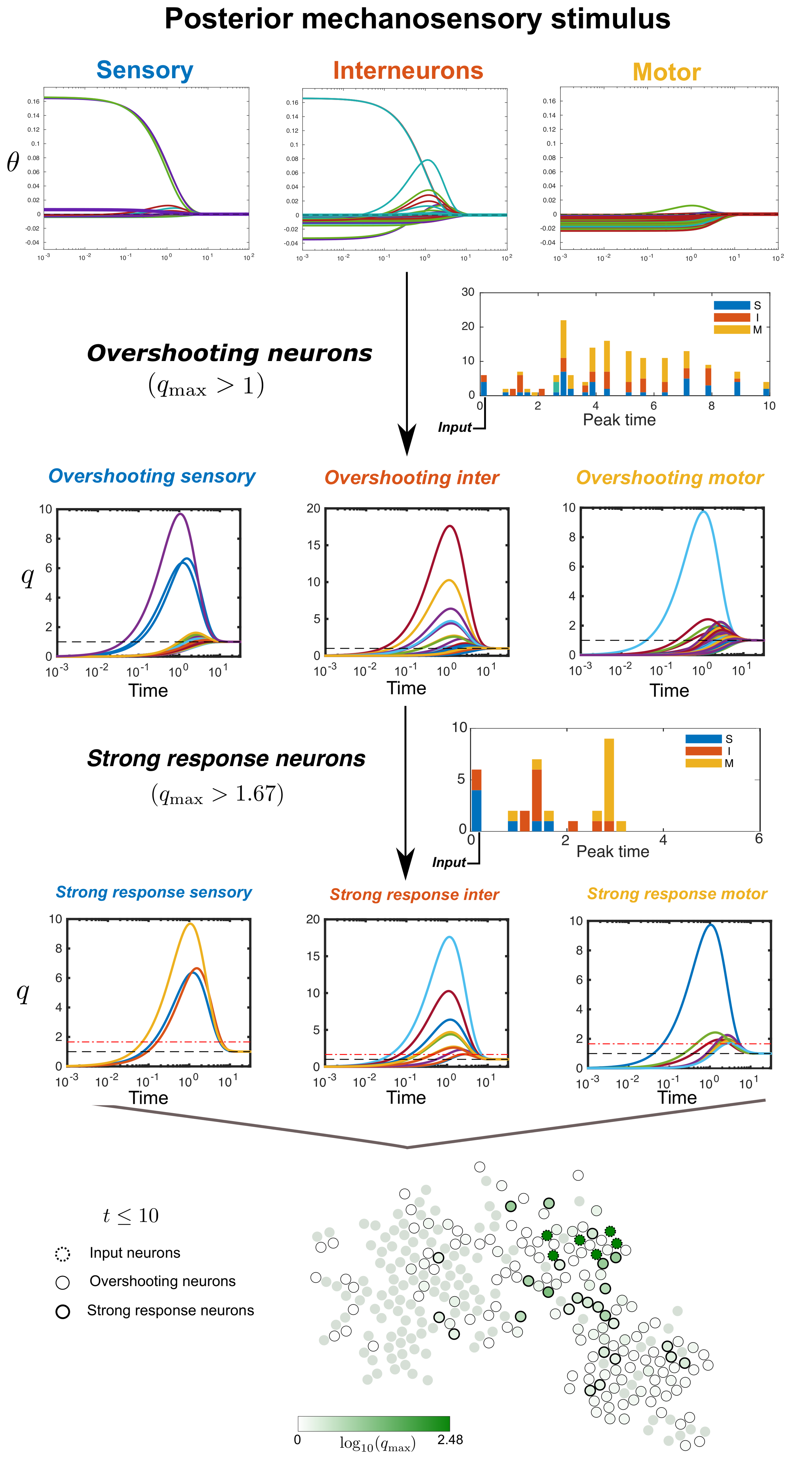

Supplement: S7 Fig — For all neurons, we compute ϕi(t), i.e., the amount of signal present at each node at Markov time t. As time grows, the signal at each node converges to its stationary value πi. Hence θi(t) = ϕi(t) − πi → 0. The approach to stationarity can happen in two ways: i) the initially negative θi(t) approaches 0 from below; ii) θi(t) ‘overshoots’ before decaying towards its stationary value. We consider the signal relative to the stationary value, qi(t) = ϕi(t)/πi, and focus on neurons that overshoot (i.e., those with qmax, i: = maxt ϕi(t)/πi > 1) and we collect the times at which they reach their peak. A concise summary of the signal propagation is given by the strong response neurons with qmax,i > 5/3. Their peak-time histogram and the particular sequence of strong response neurons is characteristic of the different input-response biological scenarios, as well as the analyses by neuron type and flow roles. (TIF) [file pcbi.1005055.s010.tif]

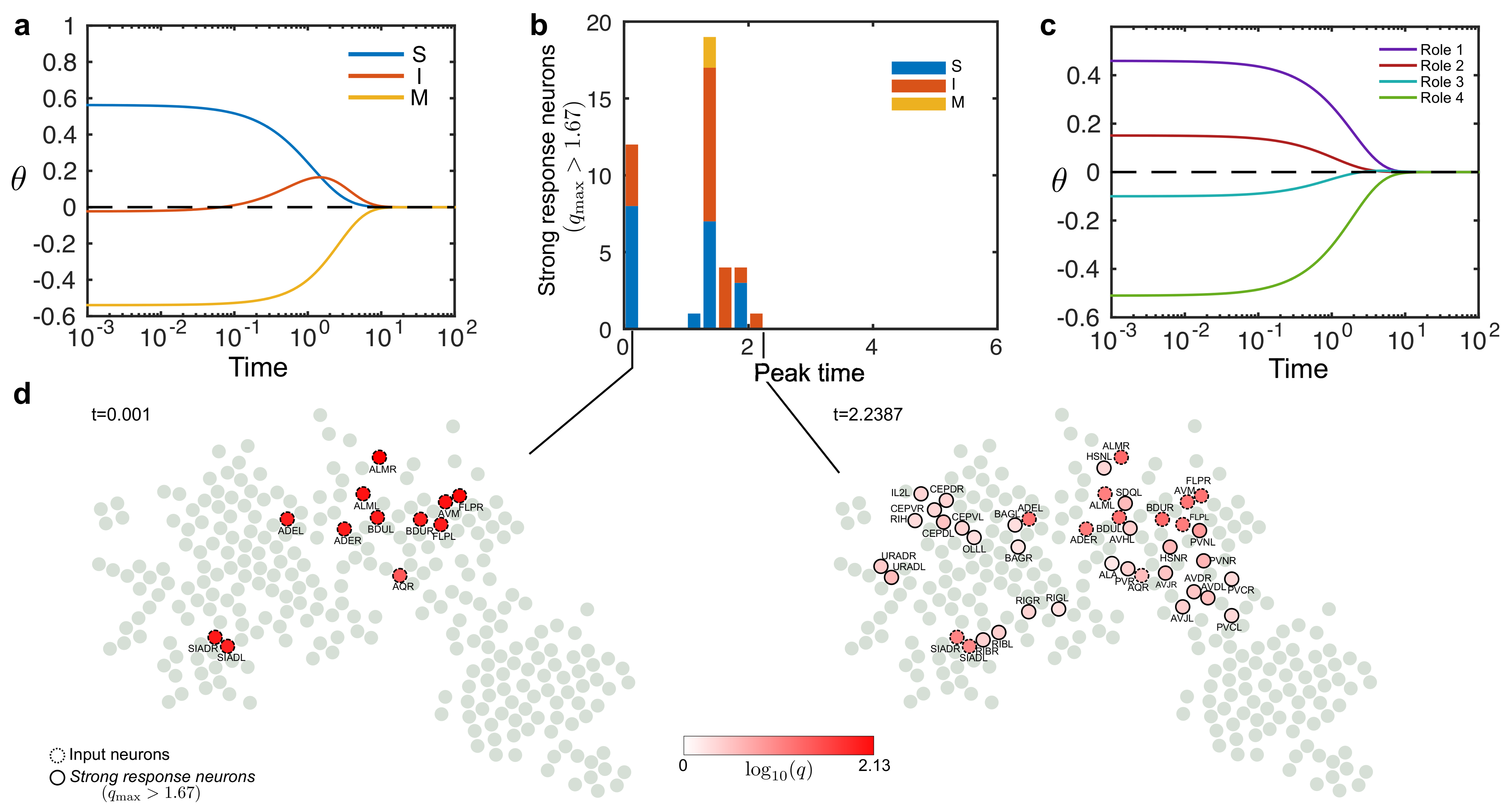

Supplement: S8 Fig — Signal propagation evolving from an initial condition localised at the mechanosensory neurons (i2). (a) As stationarity is approached (θ(t) → 0), the input propagates from sensory to motor neurons through an intermediate stage when interneurons overshoot. (b) The propagation seen as a cascade of strong response neurons (qmax,i > 1 + 2/3) with peak times concentrated around two bursts. (c) The input (i2), appears localised on R1 and to a lesser extent R2 neurons. The signal diffuses somewhat quicker out of R2 than R1 neurons, but induces not collective overshoot of R3 or R4 neurons. (d) Stages of signal propagation in the network showing the strong response neurons that have peaked at each time. (TIF) [file pcbi.1005055.s011.tif]

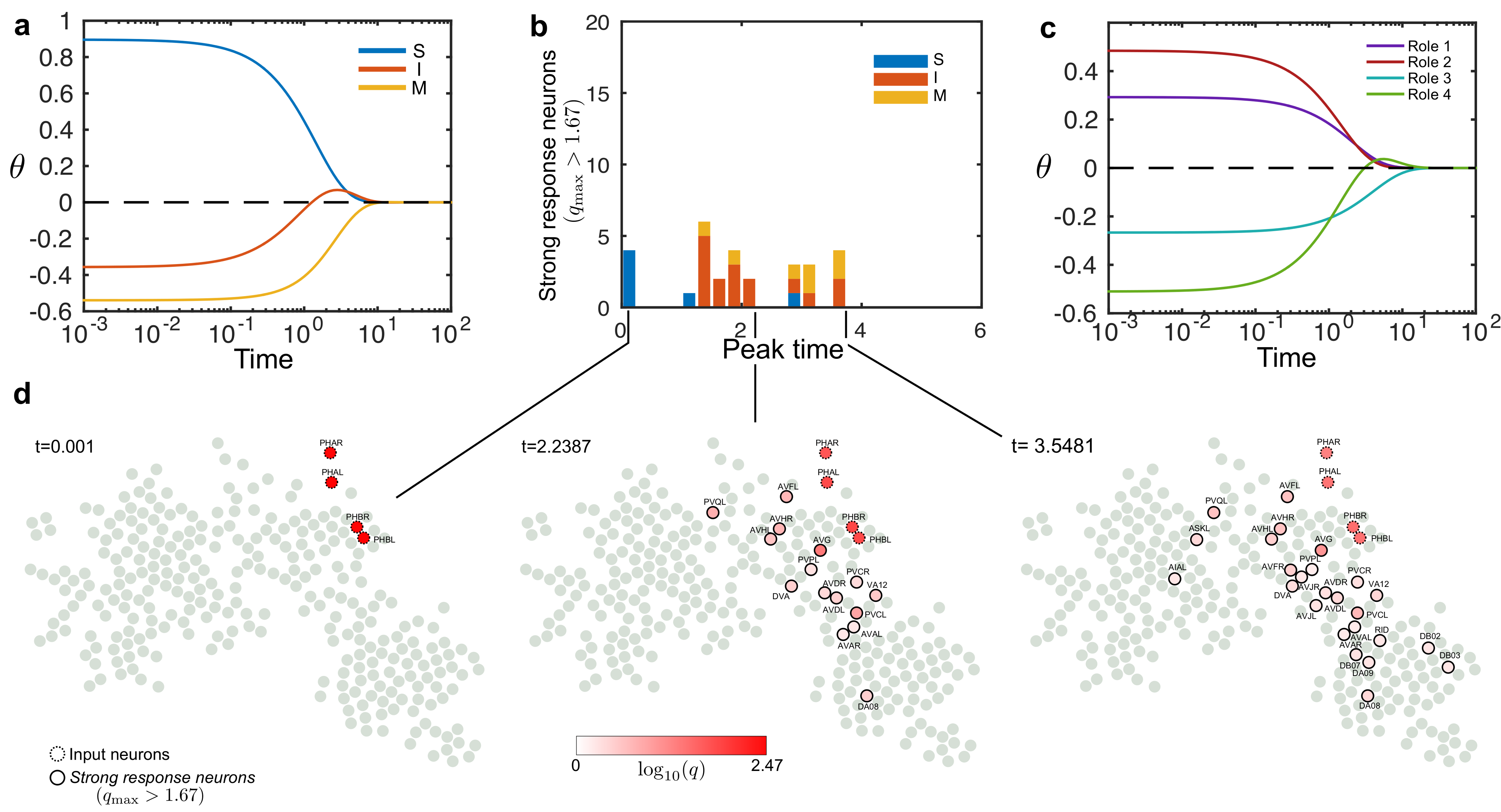

Supplement: S9 Fig — See caption of S8 Fig. (TIF) [file pcbi.1005055.s012.tif]

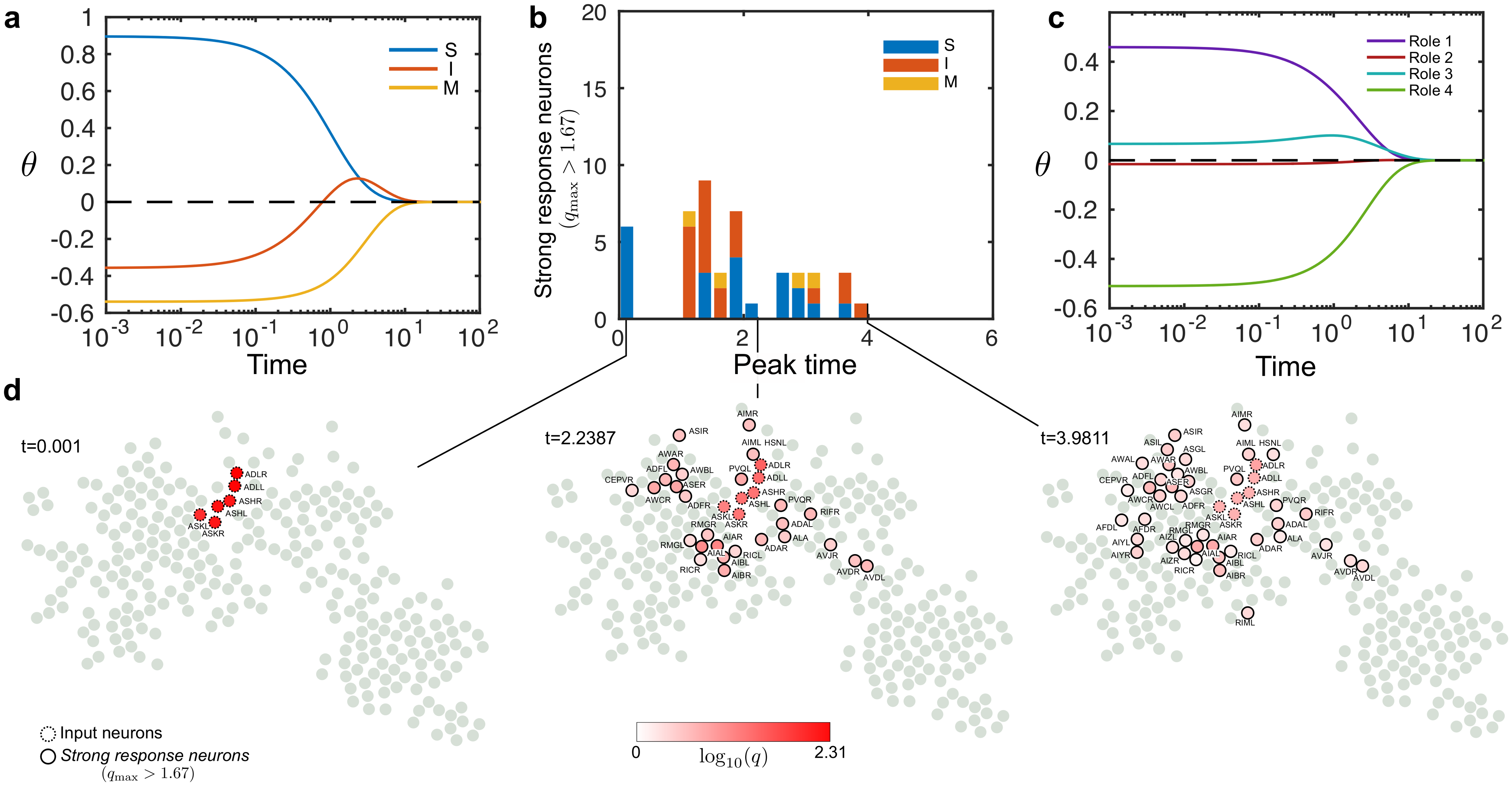

Supplement: S10 Fig — See caption of S8 Fig. (TIF) [file pcbi.1005055.s013.tif]

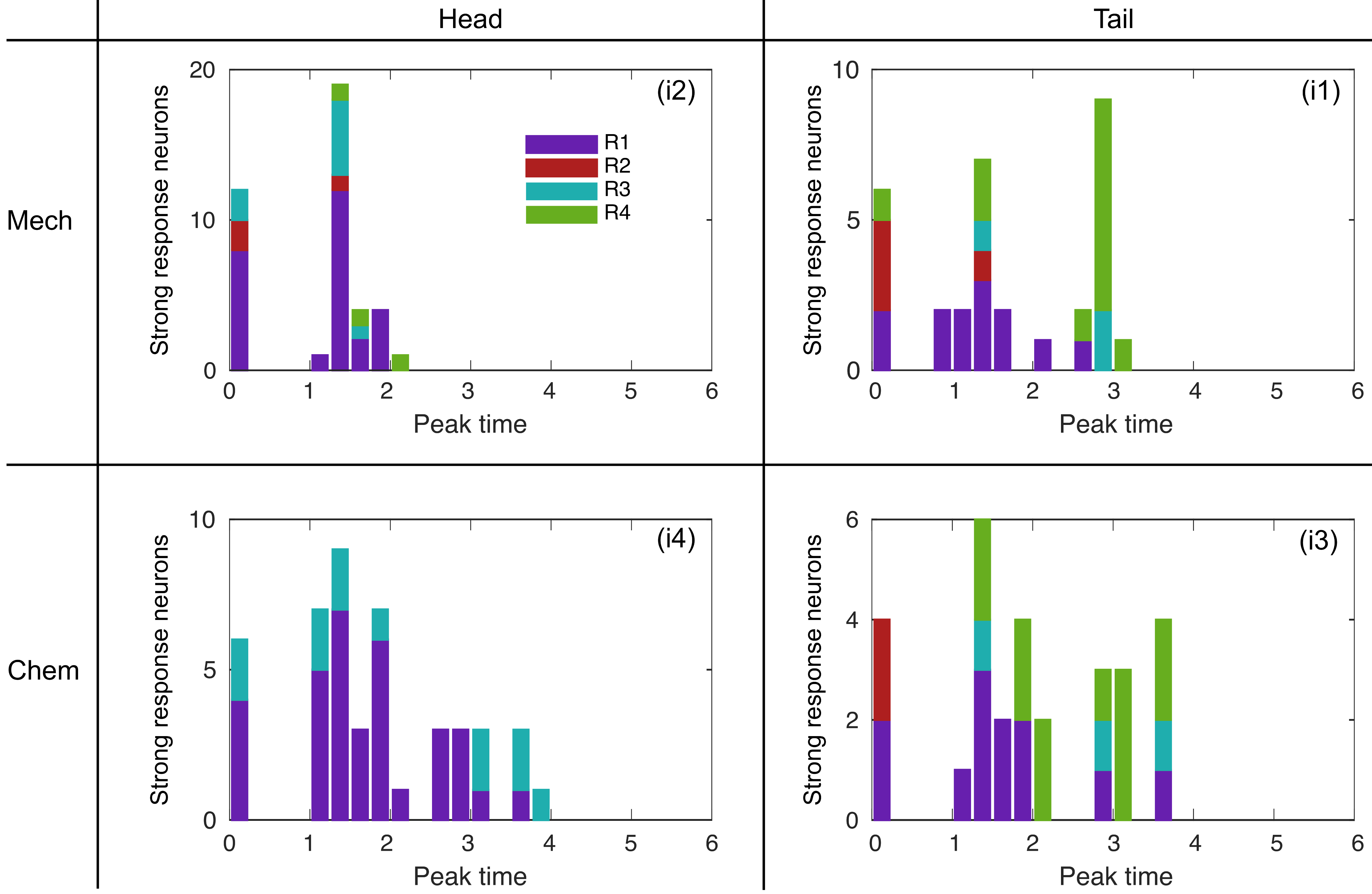

Supplement: S11 Fig — Histograms of peak times of the strong response neurons in the four biological scenarios from the perspective of flow roles. The tail inputs (i1) and (i3) induce strong responses on neurons spreading from R2 to R1 and finally to R4. On the other hand, the head inputs induce strong responses on neurons heavily based on R1 spreading downwards to R3. (TIF) [file pcbi.1005055.s014.tif]

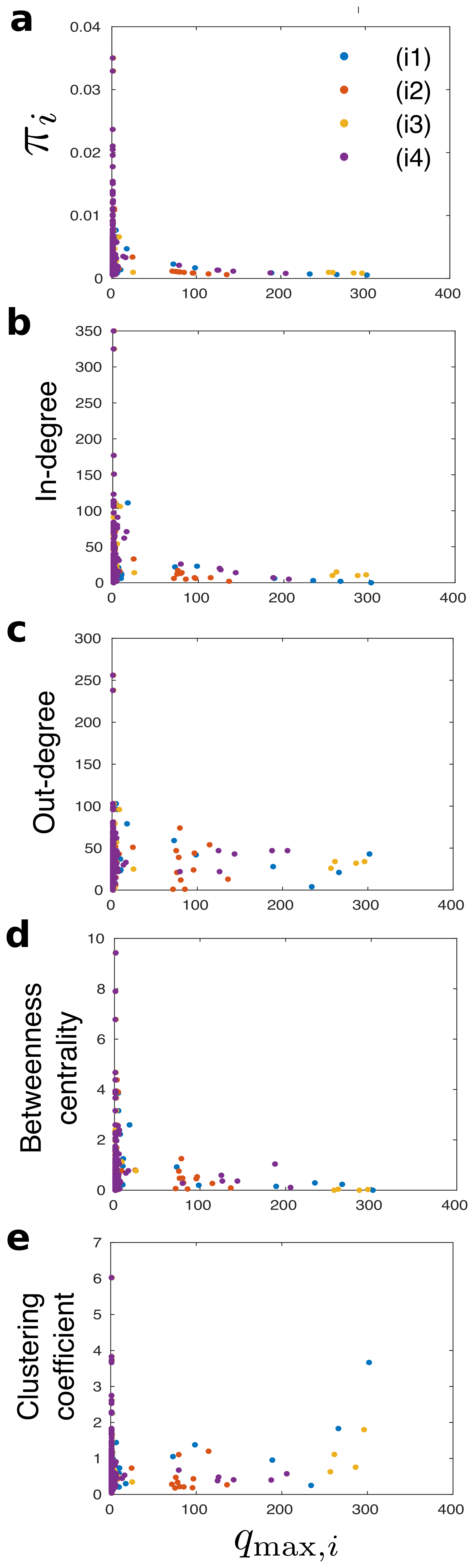

Supplement: S12 Fig — The maximum overshoot of each neuron qmax, i for each of the four biological scenarios (i1)–(i4) is plotted against the following measures of the corresponding neuron: a, stationary flow distribution π (PageRank); b, in-degree; c, out-degree; d, betweenness centrality; and e, local clustering coefficient. There is no manifest correlation between the overshooting qmax, i and any of those centrality scores or the local clustering coefficient. (TIF) [file pcbi.1005055.s015.tif]
